# Sumac (*Rhus coriaria L.*) and Human Metabolic Health: A Systematic Review and Meta‐Analysis

**DOI:** 10.1002/edm2.70135

**Published:** 2025-12-02

**Authors:** Ali Jafari, Bahare ParsiNezhad, Minoo AhmadianMoghaddam, Motahareh Hasani, Niloufar Rasaei, Ali Saeedi‐Boroujeni, Gholamreza Roshandel, Sima Besharat, Mahla Chambari, Alireza Alaghi

**Affiliations:** ^1^ Student Research Committee, Department of Community Nutrition, Faculty of Nutrition Sciences and Food Technology, National Nutrition and Food Technology Research Institute Shahid Beheshti University of Medical Sciences Tehran Iran; ^2^ Systematic Review and Meta‐Analysis Expert Group (SRMEG) Universal Scientific Education and Research Network (USERN) Tehran Iran; ^3^ Student Research Committee, Department of Nutrition, School of Health Golestan University of Medical Sciences Gorgan Iran; ^4^ Student Research Committee Lorestan University of Medical Sciences Khorramabad Lorestan Iran; ^5^ Department of Nutrition, School of Health Golestan University of Medical Sciences Gorgan Iran; ^6^ Food, Drug, Natural Products Health Research Centre GolestanUniversity of Medical Sciences Gorgan Iran; ^7^ Micronutrient Research Center, Research Institute for Endocrine Sciences Shahid Beheshti University of Medical Sciences Tehran Iran; ^8^ Department of Basic Medical Sciences, Faculty of Medicine Abadan University of Medical Sciences Abadan Iran; ^9^ Golestan Research Center of Gastroenterology and Hepatology, Jorjani Clinical Sciences Research Institute Golestan University of Medical Sciences Gorgan Iran; ^10^ Department of Food Science and Nutrition, Faculty of Applied Sciences UCSI University Kuala Lumpur Malaysia; ^11^ Faculty of Medicine Navoi State University Navoi Uzbekistan

**Keywords:** anthropometric factor, glycemic profile, lipid profile, meta‐analysis, metabolic factor, sumac

## Abstract

**Background:**

*Rhus coriaria L.* (Anacardiaceae), known as Sumac, is a commonly used spice, which is rich in various classes of phytochemicals including flavonoids, tannins, polyphenolic compounds, organic acids, and may be beneficial for cardiovascular disease risk factors. This comprehensive systematic review and meta‐analysis aimed to assess the impact of Sumac supplementation on cardiovascular disease risk factors, including anthropometric measures, glycemic profile, inflammatory markers, and lipid profile. Additionally, we explored the dose‐response relationship and optimal duration of Sumac supplementation for its effects on cardiovascular risk factors.

**Methods:**

Relevant randomized clinical trials were identified through electronic database searches (PubMed, Scopus, Web of Science, CENTRAL, and EMBASE) up to March 2025. The Cochrane risk‐of‐bias tool was employed to evaluate study quality. Standardized mean differences (SMDs) in changes between intervention and placebo groups were calculated. A random‐effects model, meta‐regression, and non‐linear modeling explored heterogeneity, dose‐response relationships, and the overall impact of Sumac supplementation.

**Results:**

Fifteen trials, with interventions ranging from 4 to 12 weeks and involving 917 participants, were included. Sumac supplementation demonstrated significant improvements in glycemic and lipid profile. Conversely, no significant effects were observed on other cardiovascular risk factors.

**Conclusions:**

Sumac supplementation improved lipid profile, and glycemic parameters suggesting potential benefits in addressing cardiovascular risk factors, despite no significant effects on inflammatory parameters.

## Graphical abstract

Sumac (*Rhus coriaria L*.) supplementation enhances glycemic control and lipid metabolism, indicating potential cardiometabolic benefits in reducing cardiovascular disease risk factors.

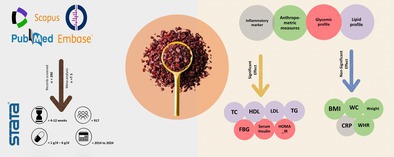

**Abbreviations**
BMI: body mass index
CI: confidence interval
CVDs: cardiovascular diseases
FBG: fasting blood glucose
HDL: high‐density lipoprotein
HOMA‐IR: homeostatic model assessment of insulin resistance
hs‐CRP: high‐sensitivity C‐reactive protein
LDL: low‐density lipoprotein
MetS: Metabolic syndrome
NAFLD: Non‐alcoholic fatty liver disease
PCOS: polycystic ovary syndrome
RCTs: randomised controlled trials
SD: standard deviations
SE: standard error
SMD: standardised mean difference
T2DM: type 2 diabetes mellitus
TC: total cholesterol
TG: triglycerides
WC: waist circumference
WHR: waist‐to‐hip ratio

## 1 Background

Cardiovascular diseases encompass a wide range of heart and blood vessel‐related conditions. Historically, they were defined as a combination of high blood glucose, high blood pressure, and gout [1]. However, contemporary understanding now includes factors such as diabetes, disturbances in lipid profiles, and elevated blood pressure [2]. These risk factors encompass disturbances in lipid profiles, notably characterised by elevated low‐density lipoprotein (LDL) cholesterol levels, a primary atherosclerosis risk factor [3]. Additionally, oxidative stress and inflammation, measured by biomarkers like interleukin‐6 and C‐reactive protein (CRP), play a significant role in atherosclerotic plaque formation and cardiovascular diseases [4]. Recent research has indicated a potential link between abdominal obesity and cardiovascular diseases through increased expression of pro‐inflammatory adipokines and reduced anti‐inflammatory adipokines [5]. High blood pressure and elevated homocysteine levels, associated with fasting blood glucose elevation, are also recognised as other cardiovascular disease risk factors [6].

Recent studies have underscored the promising potential of medicinal plants and their bioactive compounds as valuable resources for preventing and managing various health issues [7, 8]. Medicinal plants may offer effective treatments or preventive measures for numerous cardiovascular and metabolic conditions [9]. Moreover, specific plants exhibit anti‐inflammatory effects and may impact atherogenic indices and cardiometabolic parameters, offering potential mechanisms of action in addressing cardiovascular disease factors [10, 11].

*Rhus coriaria L*., commonly known as Sumac, belongs to the Anacardiaceae family. *Rhus coriaria L*. is a shrub native to the Mediterranean and Middle Eastern regions, including parts of Iran, where it is widely used traditionally. This clarifies its cultural and research prominence without implying exclusivity to Iran [12]. Sumac is a medicinal spice recognized for its multifaceted properties, including antilipidemic, antifibrogenic, anti‐inflammatory, antidiabetic, antioxidant, anti‐ischemic, antithrombotic, anti‐hypertensive, and hypoglycemic effects, as evidenced by human and animal studies [13, 14, 15, 16]. These effects may be the result of its antioxidant properties via flavonoids and tannins, along with anti‐lipase activity that inhibits lipid absorption. Furthermore, recent studies have suggested that Sumac may possess anti‐obesity effects through its antioxidant and anti‐lipase properties [17]. Oxidative stress can promote obesity by increasing the differentiation and proliferation of fat cells and inducing the deposition of white adipose tissue [18]. Sumac has been shown to possess anti‐lipase properties, potentially inhibiting pancreatic lipase enzyme secretion and thereby suppressing fat absorption in the intestine [19]. Nevertheless, studies evaluating the effects of Sumac powder supplementation on anthropometric indices have yielded contradictory results. While some studies have demonstrated the potential of Sumac powder supplementation to improve weight and body mass index (BMI) [13, 20, 21], others have failed to find significant effects on anthropometric measures [22, 23].

In addition to its reported clinical effects, the cardiometabolic benefits of sumac may be attributed to its rich phytochemical composition [24]. Over 200 bioactive compounds have been isolated from *Rhus coriaria* including phenolic acids, flavonoids, tannins, anthocyanins, terpenoids, and other antioxidant molecules [25]. These compounds contribute to its lipid‐lowering and anti‐inflammatory properties through mechanisms such as scavenging reactive oxygen species (ROS), enhancing endothelial nitric oxide synthase (eNOS) activity, and modulating inflammatory pathways including TNF‐α and cyclooxygenase (COX) [26]. Experimental evidence has shown that sumac extracts can reduce myocardial injury, improve endothelial function, and regulate lipid parameters including HDL, LDL, and triglycerides, thereby supporting its use as a cardioprotective agent [27].

However, existing evidence from multiple randomised clinical trials on the effects of Sumac is heterogeneous. Although several systematic and meta‐analytic reviews [17, 28, 29, 30, 31] have been conducted to evaluate the pharmacological properties, biological activities, medicinal uses, and nutritional aspects of Sumac, these studies are neither comprehensive nor specifically focused on cardiovascular risk factors. Furthermore, they have not examined the appropriate dosage and duration of Sumac consumption across different age groups. Since their publication, several new clinical trials have emerged [21, 23, 32]. Therefore, to address this knowledge gap, this systematic review and comprehensive meta‐analysis aim to investigate the impact of Sumac on cardiovascular risk factors and determine the optimal dosage and duration of consumption based on a GRADE assessment.

## 2 Methods

### 2.1 Protocol and Registration

The development of this extensive assessment has advanced by utilizing a predetermined methodology in accordance with the instructions and recommended specifics provided by the checklist of the 2015 Preferred Reporting Items for Systematic Reviews and Meta‐Analyses (PRISMA) guidelines [33].

The extensive assessment and meta‐analysis were officially registered in PROSPERO (International Prospective Register of Systematic Reviews), a highly regarded database for comprehensive assessments. The registration number CRD42023467804 was acquired to ensure transparency and adherence to the assessment process. Additionally, this research followed the principles outlined in the Declaration of Helsinki. Ethical approval for the research was obtained from the Ethics Committee of Golestan University of Medical Sciences (IR.GOUMS.REC.1402.346).

### 2.2 Search Strategy and Study Selection

A comprehensive evaluation was conducted to locate articles published from the beginning to March 2025. The evaluation encompassed the Cochrane Central Register of Controlled Trials (CENTRAL), Web of Science, Medline (http://www.ncbi.nlm.nih.gov/pubmed), Scopus, and EMBASE electronic databases. Additional databases such as ProQuest, which primarily indexes dissertations, theses, and select evidence‐based collections (e.g., Health Research Premium Collection), were excluded because they are less focused on primary peer‐reviewed RCTs and more likely to overlap with content from the selected sources without adding unique, relevant trials for this topic. In order to ensure comprehensiveness, supplementary sources including prominent journals and the reference lists of all pertinent research articles, meta‐analyses, and review publications pertaining to sumac were manually scrutinized to identify any additional qualified studies.

The main area of interest revolved around the variable of Sumac supplementation. The main objective was to examine the alterations in various biomarkers including body mass index (BMI), waist circumference (WC), weight, waist‐to‐hip ratio (WHR), fasting blood glucose (FBG), homeostatic model assessment of insulin resistance (HOMA‐IR), serum insulin, high‐sensitivity C‐reactive protein (hs‐CRP), high‐density lipoprotein (HDL), low‐density lipoprotein (LDL), total cholesterol (TC), and triglycerides (TG). These parameters were assessed as the main outcomes following the administration of Sumac supplementation in comparison with the placebo group.

The purpose of the literature search strategies was to identify all relevant Randomised Controlled Trials (RCTs) conducted on human subjects through the use of specific keywords and text words, namely ‘Sumac’ and ‘clinical trial’. The terms utilised in this study were obtained from the EMTREE and Medical Subject Headings (MeSH) tags of the PubMed database. A combination of these terms was employed to develop an appropriate electronic search strategy. The search approach was modified for PubMed, Web of Science, EMBASE, Scopus, and CENTRAL by incorporating their respective subject headings instead of the MeSH subject headings (Table S1).

### 2.3 Inclusion/Exclusion Criteria and Data Extraction

The meta‐analysis included studies that met specific criteria. These criteria considered factors such as the incorporation of primary studies consisting of RCTs in the systematic review, the requirement for the included RCTs to be at least single‐blind and to have either parallel or crossover designs, the presence of concurrent control groups in the included studies, and the publication of the studies in the English language. Conversely, studies were excluded from the analysis if they did not meet certain criteria. These criteria included the exclusion of methods other than RCTs conducted on human subjects, the exclusion of study types such as review articles, case studies, case series, observational studies (including cross‐sectional, case–control, and cohort studies), and experimental studies involving animals or in vitro. Additionally, proceedings, editorials/commentaries, letters, and reports were excluded if they lacked sufficient data on baseline or follow‐up cardiometabolic factors, including anthropometric measures, glycemic profile, inflammatory markers, and lipid profile in each group. Furthermore, studies that reported the combination of Sumac with other substances were also excluded.

Two reviewers (A.S. and A.A.) conducted the initial investigation independently and acquired the complete texts of the studies that fulfilled the inclusion criteria, and any disagreement was solved by the third author (A.J.), who made the final decision. These texts were subsequently evaluated to determine their suitability. The management of the collected data was accomplished through the utilisation of EndNote X7 software. The search outcomes from different databases were merged, and any duplications were eliminated. In accordance with the PRISMA guidelines, the selection process for this systematic review was carried out. The eligible articles were thoroughly examined, and the pertinent information was extracted by employing a standardised data extraction form developed by the primary reviewers (A.J. and G.R.). Two independent researchers (B.P. and M.A.) conducted data extraction from the included studies, encompassing a range of pertinent variables including author information, study year, country, study design, population details, demographic characteristics, intervention and control specifics, and outcome measures. In instances of discord, resolution efforts were undertaken through discussion. If consensus proved elusive, the matter was escalated to the project supervisor (A.J) to ensure a unified interpretation.

### 2.4 Methodological Quality Assessment and Evaluation of the Strength of Evidence

Two reviewers (M.H. and S.B.) conducted a thorough evaluation of the methodological quality of the trials included in the study, utilising the Cochrane criteria. In case of any disagreements, the authors will resolve them through discussion. If consensus cannot be reached, they will involve a third reviewer (A.J.). The criteria employed to assess each individual trial were as follows: adequacy of sequence generation, allocation concealment, blinding, addressing of dropouts (incomplete outcome data), selective outcome reporting, and identification of other potential sources of bias.

To evaluate and summarise the body of evidence related to each outcome analysed in the meta‐analysis, we employed the Grading of Recommendations, Assessment, Development, and Evaluation (GRADE) framework. This framework categorises evidence obtained from systematic reviews and meta‐analyses into four distinct categories: “high,” “moderate,” “low,” or “very low.” The quality of the reported effect size can be upgraded or downgraded based on the study's design, as well as other methodological and statistical limitations. Two independent reviewers (A.J. and N.R.), with one author (G.R.) acting as an adjudicator in case of discrepancies, took into consideration various factors for each outcome to assess the certainty of the estimate. These factors included study design, risk of bias, consistency, precision, directness, presence of a substantial effect, dose–response gradient, and publication bias.

### 2.5 Statistical Analysis

The statistical analysis was carried out utilizing the Stata software, version 17.0, developed by Stata Corp in College Station, Texas, for the purpose of conducting a meta‐analysis. Our analysis involved extracting the means of pre‐ and post‐treatment, standard deviations, and participant numbers from both the intervention and placebo groups for various clinical outcomes, including anthropometric measures, blood pressure, glycemic profile, inflammatory markers, lipid profile, oxidative stress parameters, and leptin. In order to evaluate the impact of Sumac supplementation, we assessed the standardized mean differences (SMDs) in changes between the intervention and placebo groups.

Due to the varying populations and settings among the included randomised controlled trials (RCTs), a random effects model was employed to calculate the overall effect based on the SMDs, utilising the DerSimonian–Laird weighting method [34]. When standard deviations (SDs) were not provided for changes but only for baseline and the end of the trial, the SD for net change was calculated using the Follmann method [35] with an assumed correlation coefficient (R) of 0.5. If the standard error (SE) was given, the SD was assigned using the formula SD = SE × sqrt(n), where n represented the sample size in each group. The standard deviations (SDs) of the change difference were computed using the formula: SD = square root [(SD pre‐treatment)^2^ + (SD post‐treatment)^2^ − (2R × SD pre‐treatment × SD post‐treatment)], with an assumed correlation coefficient (*R*) of 0.5 [36].

The *I*‐square (*I* ^2^) statistic was utilized to assess the heterogeneity between studies. Furthermore, we computed the prediction interval to evaluate the clinical significance of the observed variations across studies. Subgroup analyses were conducted to investigate the impact of Sumac on clinical outcomes, taking into consideration relevant study characteristics such as study population, duration of follow‐up, and Sumac dosage as potential sources of heterogeneity. Meta‐regression was employed to examine the potential influence of Sumac dosage (expressed in grams per day) and duration on risk factors associated with cardiovascular diseases (CVDs).

Additionally, a non‐linear model was employed to integrate dose–response data from various studies in order to evaluate the effect of Sumac supplementation on CVD risk factors [37]. In order to explore the reliance of the meta‐analysis results on individual studies, we conducted a leave‐one‐out analysis by recalculating the meta‐analysis statistic after excluding one study at a time.

## 3 Results

### 3.1 Study Selection

**FIGURE 1:**
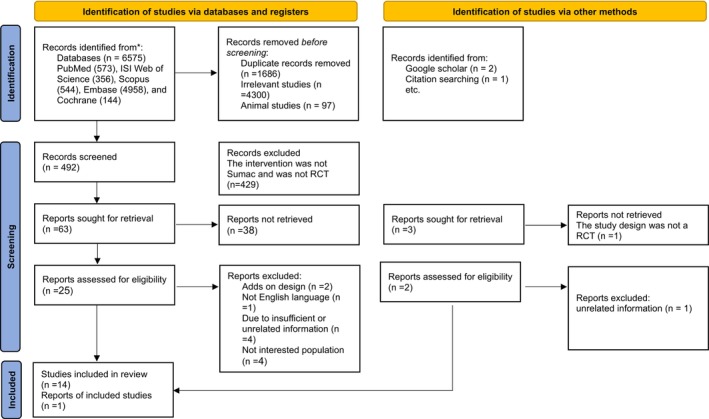
Flowchart of study selection for inclusion trials in the systematic review.

The selection process of the included studies is presented in Figure 1. A database search resulted in identifying a total of 6575 studies, including PubMed (*n* = 573), ISI Web of Science (*n* = 356), Scopus (*n* = 544), Embase (*n* = 4958) and Cochrane Library (*n* = 144). A total of 1686 duplicated studies were excluded, and 492 studies were screened based on title and abstract. After screening, 429 irrelevant studies were excluded, and 25 full‐text studies were considered. In the end, 11 studies were excluded due to reporting non‐desired outcomes and one study was added by manual search. Table S2 shows the studies that were excluded from the full‐text review and the reasons for exclusion. As a result, 15 studies with 917 participants were included in the systematic review and meta‐analysis [13, 14, 15, 20, 21, 22, 23, 32, 38, 39, 40, 41, 42, 43, 44].

### 3.2 Study Characteristics

**FIGURE 2:**
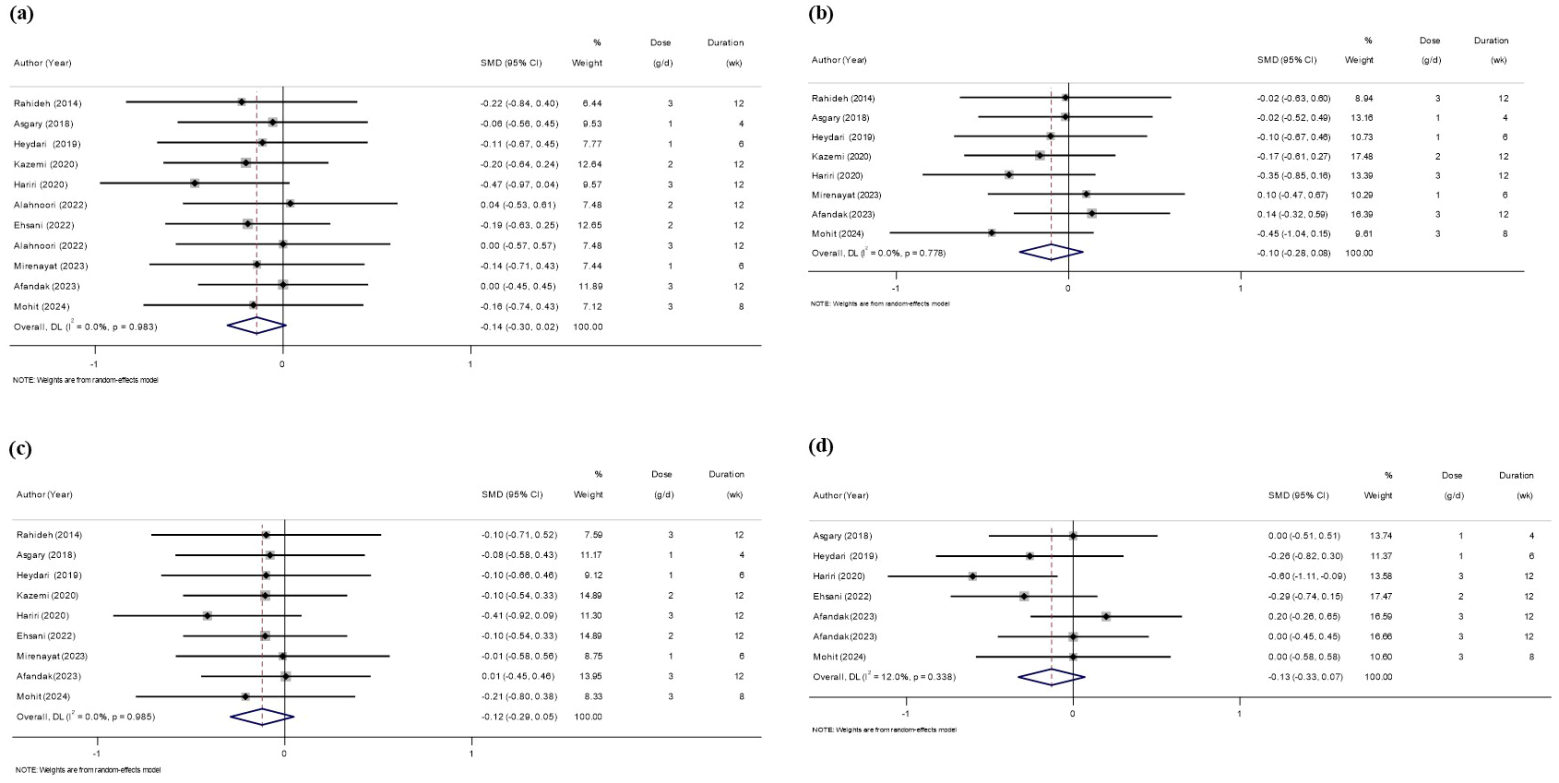
Forest plot of the effects of Sumac supplement on anthropometric measures (a, BMI; b, WC; c, Weight; d, WHR).

The characteristics of included studies are presented in Table 1. The SMD and 95% CI of BMI, WC, weight, WHR, FBG, HOMA‐IR, serum insulin, hs‐CRP, HDL, LDL, TC, TG, and their changes in comparison with the placebo group are presented in Figure 2 respectively. The studies were published between 2014 and 2024 and were carried out in Iran. In the intervention group, the mean age was between 24.66 and 52.30 years old. The Sumac dose was between 1 g/d and 6 g/d. The duration of intervention was between 4 and 12 weeks. The sample size in the intervention group was between 22 and 68. Three studies included only females [13, 21, 32] and the rest of the studies included both genders. Studies included participants with primary hyperlipidemia [15], dyslipidemia [39], haemodialysis [41], metabolic syndrome (MetS) [23, 43], non‐alcoholic fatty liver disease (NAFLD) [42, 43, 44], polycystic ovary syndrome (PCOS) [32], type 2 diabetes mellitus (T2DM) [14, 22, 38], and overweight or obese [13, 21, 39] subjects.

**TABLE 1:** Characteristic of included studies in the meta‐analysis.

Studies, year (Ref.)	Country	Study design	Participant	Sample size (sex)	Sample size (INT/CON)	Trial duration (week)	Means age (INT/CON)	Dose of supplement (g/d)	Type of supplement (INT/CON)	Outcomes
Shidfar et al. 2014 [14]	Iran	DB, RCT	T2DM	41 (B)	22/19	12	46.10/47.50	3	Sumac powder/placebo	FBG
Rahideh et al. 2014 [38]	Iran	DB, RCT	T2DM	41 (B)	22/19	12	46.10/47.50	3	Sumac powder/placebo	Weight, BMI, WC, HOMA‐IR, hs‐CRP, Serum Insulin
Ardakani et al. 2016 [22]	Iran	RCT	T2DM	58 (B)	30/28	12	52.30/51.61	6	Sumac powder+ low‐fat yoghurt/low‐fat Sumac‐free yoghurt	FBG, Serum Insulin
Asgary et al. 2018 [20]	Iran	TB, RA, crossover	Dyslipidemia	60 (B)	30/30	4	45.62/49.26	1	Sumac capsule/placebo	WC, Weight, BMI, WHR SBP, DBP, TC, TG, LDL, HDL, FPG
Hajmohammadi et al. 2018 [15]	Iran	DB, RCT	Primary Hyperlipidemia	70 (B)	34/36	6	45.32/42.53	1	Rhus POWDER/placebo	TG, TC LDL, HDL
Heydari et al. 2019 [39]	Iran	DB, RCT	Overweight or obese	49 (B)	25/24	6	45.16/43.13	1	Sumac capsule/placebo	Weight, WC, WHR, BMI, FBG, HOMA, Serum Insulin
Kazemi et al. 2020 [40]	Iran	DB, RCT	NAFLD	80 (B)	40/40	12	41.80/41.40	2	Sumac powder/placebo	Weight, BMI, WC, FBG, HOMA‐IR, hs‐CRP, Serum Insulin
Hariri et al. 2020 [13]	Iran	DB, RCT	Overweight or Obese	62 (F)	31/31	12	42.19/44.03	3	Sumac capsule + restricted calorie diet/placebo + restricted calorie diet	Weight, BMI, WC, WHR, Serum Insulin
Ehsani et al. 2022 [42]	Iran	DB, RCT	NAFLD	80 (B)	40/40	12	41.80/41.40	2	Sumac powder/placebo	Weight, BMI, WHR, TC, LDL, HDL, TG
Alahnoori et al. 2022 [41]	Iran	TB, RCT	Haemodialysis	105 (B)	68/37	12	35–70/35–70	3	Sumac powder/placebo	TC, LDL, HDL TG, BMI
Hajhashemy et al. 2023 [43]	Iran	TB, RCT, crossover	MetS	47 (B)	24/23	6	20–70/20–70	1	Sumac capsule/placebo	FBG, Serum Insulin, HOMA‐IR, Hs‐CRP
Afandak et al. 2023 [32]	Iran	DB, RCT	PCOS	75 (F)	39/36	12	24.66/25.33	3	Sumac powder/placebo	Weight, BMI, WC, WHR, WHtR, FBG, Serum Insulin Hs‐CRP, HDL, TC, TG, LDL
Hariri et al. 2023 [21]	Iran	DB, RCT	Overweight or obese	57 (F)	28/29	12	42.89/45.13	3	Sumac capsule + restricted calorie diet/placebo + restricted calorie diet	FBG, HOMA‐IR,
Mirenayat et al. 2023 [23]	Iran	TB, RCT, crossover	MetS	47 (B)	23/24	6	20–70/20–70	1	Sumac capsule/placebo	Weight, BMI, WC, FBG, TG TC, HDL, LDL
Mohit et al. 2024 [44]	Iran	DB, RCT	Overweight or Obese	45 (B)	23/22	8	45/46	3	Sumac capsule/Placebo	Weight, BMI, WC, WHR, FBG, Serum Insulin, HOMA‐IR, Hs‐CRP, HDL, TC, LDL

Abbreviations: B, both sex; BMI, body mass index; CON, control group; DB, double‐blinded; F, female; FBG, fasting blood glucose; HDL, high‐density lipoprotein; HOMA‐IR, homeostatic model assessment of insulin resistance; hs‐CRP, high‐sensitivity C‐reactive protein; INT, intervention group; LDL, low‐density lipoprotein; MetS, metabolic syndrome; NAFLD, non‐alcoholic fatty liver disease; PCOS, polycystic ovary syndrome; RCT, randomised controlled trial; T2DM, type 2 diabetes mellitus; TB, triple‐blinded; TC, total cholesterol; TG, triglycerides; WC, waist circumference; WHR, waist‐to‐hip ratio.

### 3.3 Qualitative Data Assessment

Based on the Cochrane Risk of Bias Assessment tool, a total of two studies were considered with a high risk of bias [14, 22], four studies had a moderate risk of bias [13, 22, 38, 41] and nine studies had a low risk of bias [15, 20, 21, 23, 39, 40, 42, 43, 44] (Table 2).

**TABLE 2:** Quality of included studies in the meta‐analysis.

Study, year (Ref.)	Random sequence generation	Allocation concealment	Blinding of participants and personnel	Blinding of outcome assessment	Incomplete outcome data	Selective outcome reporting	Other sources of bias	Overall quality
Shidfar, 2014 [14]	U	U	L	H	L	L	L	Poor
Rahideh, 2014 [38]	L	L	L	L	H	L	L	Fair
Ardakani. 2016 [22]	L	H	H	H	L	L	L	Poor
Asgary, 2018 [20]	L	L	L	U	L	L	L	Good
Hajmohammadi, 2018 [15]	L	U	L	L	L	L	L	Good
Heydari, 2019 [39]	L	U	L	L	L	L	L	Good
Kazemi, 2020 [40]	L	L	L	L	L	L	L	Good
Hariri, 2020 [13]	L	L	L	H	L	L	L	Fair
Ehsani, 2022 [42]	L	L	L	L	U	L	L	Good
Alahnoori, 2022 [41]	L	L	L	L	H	L	L	Fair
Hajhashemy, 2023 [43]	L	U	L	L	L	L	L	Good
Afandak, 2023 [32]	L	L	L	H	L	L	L	Fair
Hariri, 2023 [21]	L	L	L	L	L	L	L	Good
Mirenayat, 2023 [23]	L	L	L	L	L	L	L	Good
Mohit, 2024 [44]	L	L	L	L	L	L	L	Good

Abbreviations: H, high risk of bias; L, low risk of bias; U, unclear risk of bias.

### 3.4 Effects of Sumac Supplementation on Anthropometric Measures

Figure 2 delineates the impact of Sumac supplementation on anthropometric measures in comparison with the placebo group. Meta‐analysis of 11 studies indicated no significant effect of sumac on BMI (SMD = −0.14; 95% CI: −0.30, 0.02; *p* = 0.078), with no heterogeneity (*I* ^2^ = 0%; *p* = 0.983). Similarly, no effects were observed for WC (8 studies; SMD = −0.10; 95% CI: −0.28, 0.08; *p* = 0.281; *I* ^2^ = 0%; *p* = 0.778), weight (9 studies; SMD = −0.12; 95% CI: −0.29, 0.05; *p* = 0.161; *I* ^2^ = 0%; *p* = 0.985), or WHR (7 studies; SMD = −0.13; 95% CI: −0.33, 0.07; *p* = 0.201; *I* ^2^ = 12%; *p* = 0.338).

Sensitivity analysis conducted for BMI, WC, WHR, and weight revealed that excluding any of the studies did not alter the overall findings.

Additionally, there was no evidence of publication bias for BMI (Egger's *p* = 0.70), WC (Egger's *p* = 0.70), weight (Egger's *p* = 0.76), and WHR (Egger's *p* = 0.71).

### 3.5 Effects of Sumac Supplementation on Glycemic Profile

**FIGURE 3:**
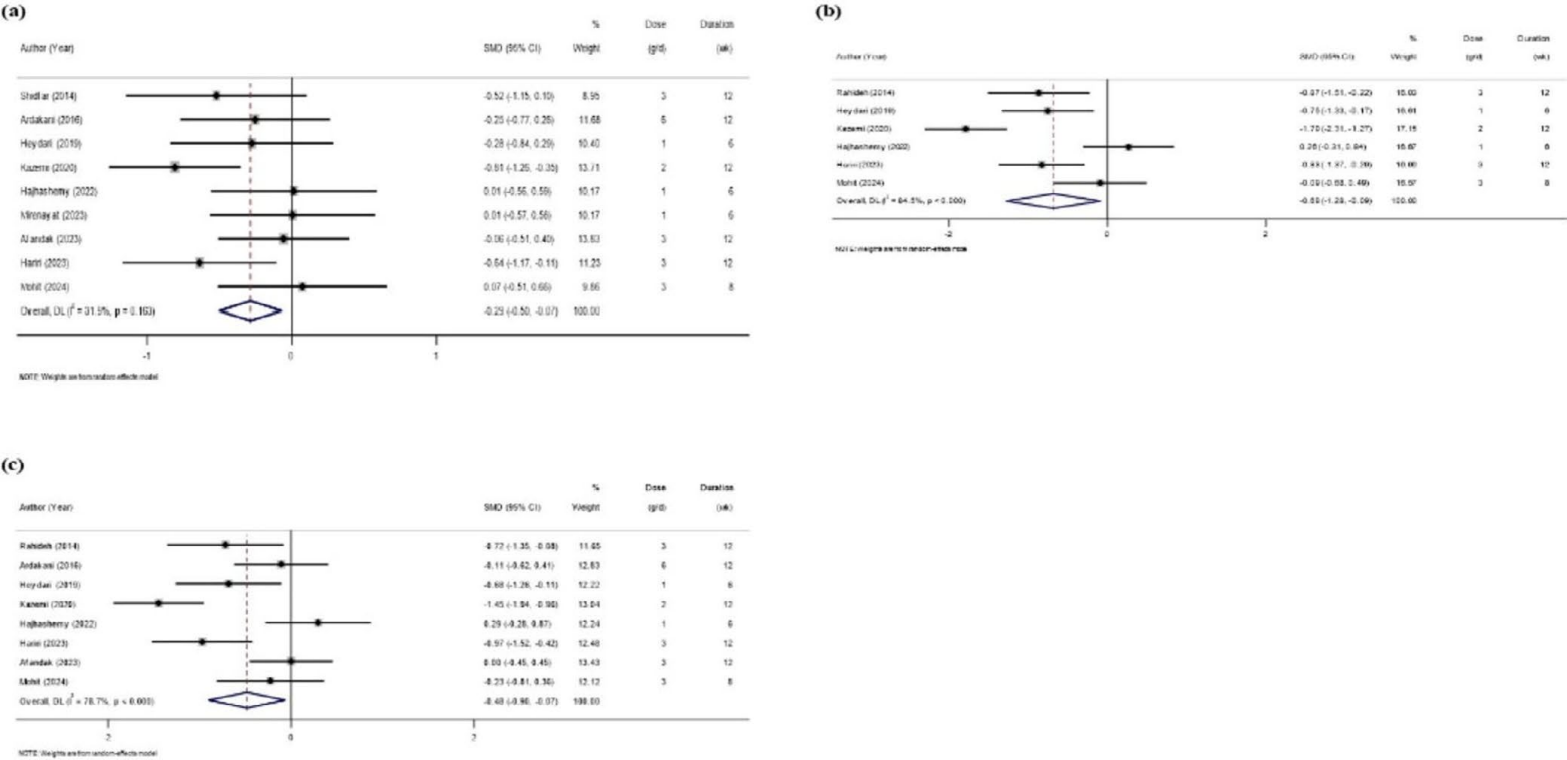
Forest plot of the effects of Sumac supplement on glycemic profile (a, FBG; b, HOMA‐IR; c, Serum insulin).

Figure 3 presents an overview of the impact of Sumac supplementation on glycemic‐related parameters. Key findings from Figure 3 show significant reductions in FBG (9 studies; SMD = −0.29; 95% CI: −0.50, −0.07; *p* = 0.010; I^2^: 31.9%; *p* = 0.163), HOMA‐IR (6 studies; SMD = −0.68; 95% CI: −1.28, −0.09; *p* = 0.024; *I* ^2^: 84.5.2%; *p* < 0.001), and serum insulin (8 studies; SMD = −0.48; 95% CI: −0.90, −0.07; *p* = 0.023; I^2^: 78.7%; *p* < 0.001).

Upon exclusion of any studies, the overall effect of Sumac on FBG level did not change significantly. After excluding the study by Hariri et al. [21] (SMD = −0.65; 95% CI: −1.38, 0.08), Rahideh et al. [38] (SMD = −0.64; 95% CI: −1.35, 0.06), and Heydari et al. [39] (SMD = −0.66; 95% CI: −1.39, 0.05), the overall effect of Sumac on HOMA‐IR significantly changed. After excluding the study by Heydari et al. [39] (SMD = −0.45; 95% CI: −0.92, 0.01), Kazemi et al. [40] (SMD = −0.33; 95% CI: −0.66, 0.00), Hariri et al. [21] (SMD = −0.41; 95% CI: −0.86, 0.04), and Rahideh et al. [38] (SMD = −0.45; 95% CI: −0.91, 0.01), the overall effect of Sumac on serum insulin changed significantly.

Additionally, there was no evidence of publication bias in the studies examining FBG (Egger's *p* = 0.29), HOMA‐IR (Egger's *p* = 0.34), and serum insulin (Egger's *p* = 0.92).

### 3.6 Effects of Sumac Supplementation on Inflammatory Marker

**FIGURE 4:**
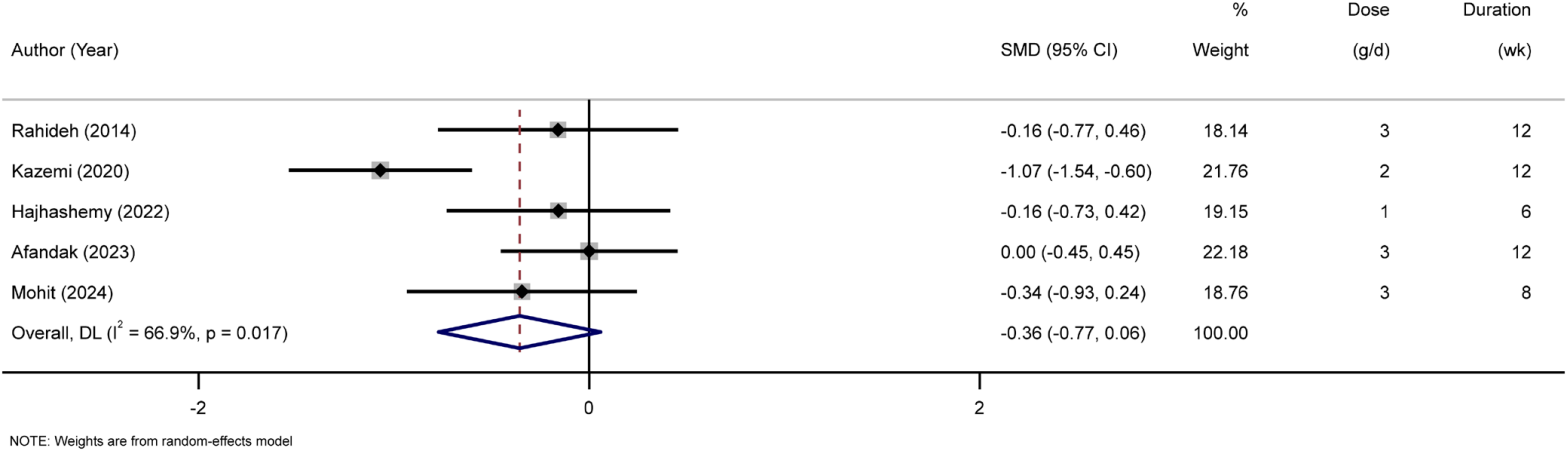
Forest plot of the effects of Sumac supplement on inflammatory markers (hs‐CRP).

Figure 4 details the impact of Sumac supplements on inflammatory factors. A total of five studies were included in the investigation of the effect of Sumac supplements on hs‐CRP compared to the placebo group. The meta‐analysis results indicated that there was no significant effect of Sumac supplements on hs‐CRP compared with the control group (SMD = −0.36; 95% CI: −0.77, 0.06; *p* = 0.092). High heterogeneity was observed among the studies (*I* ^2^: 66.9%, *p* = 0.017).

The sensitivity analysis for hs‐CRP revealed that excluding any of the studies did not significantly alter the pooled effect size.

Furthermore, there was no identified evidence of publication bias for hs‐CRP (Egger's *p* = 0.66).

### 3.7 Effects of Sumac Supplement on Lipid Profile

**FIGURE 5:**
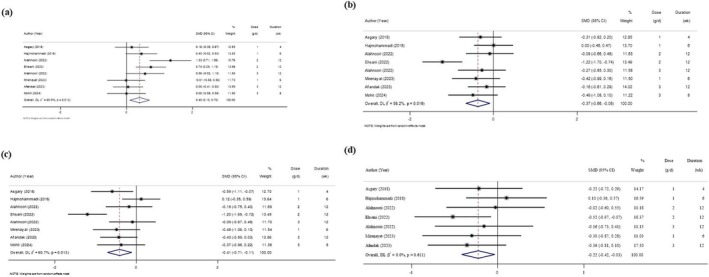
Forest plot of the effects of Sumac supplement on lipid profile (a, HDL; b, LDL; c, TC; d, TG).

Figure 5 provides an overview of the impact of Sumac supplements on the lipid profile. Key findings from Figure 5 demonstrate significant increases in HDL (8 studies; SMD = 0.40; 95% CI: 0.10, 0.70; *p* = 0.008; *I* ^2^: 60.9%; *p* = 0.012) and reductions in LDL (8 studies; SMD = −0.37; 95% CI: −0.66, −0.08; *p* = 0.011; *I* ^2^: 58.2%; *p* = 0.019), TC (8 studies; SMD = −0.41; 95% CI: −0.71, −0.11; *p* = 0.007; *I* ^2^: 60.7%; *p* = 0.01), and TG (7 studies; SMD = −0.22; 95% CI: −0.42, −0.03; *p* = 0.021; *I* ^2^: 0.0%; *p* = 0.611).

The sensitivity analysis for HDL, LDL and TC revealed that excluding any of the studies did not significantly alter the pooled effect size. Moreover, upon removing the study by Ehsani et al. [42] (SMD = −0.15, 95% CI: −0.36, 0.05) and Afandak et al. [32] (SMD = −0.19, 95% CI: −0.40, 0.01), the overall effect of Sumac on TG significantly changed.

Moreover, there was no identified evidence of publication bias for HDL (Egger's *p* = 0.62), LDL (Egger's *p* = 0.92), TC (Egger's *p* = 0.65), and TG (Egger's *p* = 0.44).

### 3.8 Subgroup Analysis

Table 3 presents a subgroup analysis based on health status, including endocrine/metabolic conditions (PCOS, T2DM, NAFLD, and MetS), and other conditions (haemodialysis, primary hyperlipidemia, and overweight or obesity), intervention duration (≥ 6 weeks and < 6 weeks), and the dosage of the Sumac supplement (< 3 g and ≥ 3 g). These subgroups were pre‐planned based on prior literature suggesting their influence on cardiometabolic responses to herbal supplements, and the existence of different groups in extracted data to find potential sources of heterogeneity.

**TABLE 3:** Description of the analysis and subgroup results of Sumac supplementation on cardiovascular disease risk factors.

	Studies (*N*)	Participant (*N*)	SMD (95% CI)	*p*	Heterogeneity		
					*p* heterogeneity	*I* ^2^	*p* between sub‐groups
*Analysis and subgroup results of Sumac supplementation on BMI*							
Overall effect	11	643	−0.14 (−0.30, 0.02)	0.078	0.983	0	
Health status							
Endocrine/metabolic conditions	6	383	−0.13 (−0.33, 0.07)	0.205	0.986	0	0.867
Other conditions	5	270	−0.15 (−0.40, 0.09)	0.216	0.692	0	
Duration of intervention (weeks)							
6 < weeks	7	442	−0.15 (−0.34, 0.03)	0.110	0.838	0	0.801
6 ≥ weeks	4	201	−0.11 (−0.38, 0.16)	0.431	0.994	0	
Sumac dosage (g/day)							
≥ 3 g	5	275	−0.16 (−0.40, 0.07)	0.175	0.688	0	0.786
< 3 g	6	368	−0.12 (−0.32, 0.08)	0.246	0.989	0	
*Analysis and subgroup results of Sumac supplementation on WC*							
Overall effect	8	459	−0.10 (−0.28, 0.08)	0.281	0.778	0	
Health status							
Endocrine/metabolic conditions	5	313	−0.001 (−0.22, 0.22)	0.994	0.905	0	0.134
Other Conditions	3	156	−0.29 (−0.61, 0.01)	0.065	0.693	0	
Duration of intervention (weeks)							
6 < weeks	4	258	−0.09 (−0.34, 0.14)	0.433	0.549	0	0.969
6 ≥ weeks	4	201	−0.10 (−0.38, 0.17)	0.457	0.594	0	
Sumac dosage (g/day)							
≥ 3 g	4	223	−0.14 (−0.42, 0.13)	0.303	0.355	0	0.668
< 3 g	4	236	−0.06 (−0.31, 0.19)	0.631	0.899	0	
*Analysis and subgroup results of Sumac supplementation on weight*							
Overall effect	9	539	−0.12 (−0.29, 0.05)	0.161	0.985	0	
Health status							
Endocrine/metabolic conditions	6	383	−0.06 (−0.26, 0.13)	0.513	0.999	0	0.324
Other Conditions	3	156	−0.25 (−0.57, 0.06)	0.113	0.705	0	
Duration of intervention (weeks)							
6 < weeks	5	338	−0.13 (−0.34, 0.07)	0.215	0.809	0	0.832
6 ≥ weeks	4	201	−0.09 (−0.37, 0.18)	0.491	0.972	0	
Sumac dosage (g/day)							
≥ 3 g	4	223	−0.17 (−0.43, 0.09)	0.200	0.672	0	0.619
< 3 g	5	316	−0.08 (−0.30, 0.13)	0.450	0.999	0	
*Analysis and subgroup results of Sumac supplementation on WHR*							
Overall effect	7	446	−0.13 (−0.33 0.07)	0.201	0.338	12	
Health status							
Endocrine/metabolic conditions	4	290	−0.03 (−0.26, 0.20)	0.801	0.496	0	0.184
Other conditions	3	156	−0.31 (−0.65, 0.03)	0.078	0.304	16	
Duration of intervention (weeks)							
6 < weeks	4	292	−0.16 (−0.49, 0.16)	0.333	0.105	51.1	0.728
6 ≥ weeks	3	154	−0.08 (−0.39, 0.23)	0.610	0.754	0	
Sumac dosage (g/day)							
≥ 3 g	4	257	−0.09 (−0.43, 0.24)	0.595	0.128	47.2	0.663
< 3 g	3	189	−0.19 (−0.47, 0.09)	0.189	0.664	0	
*Analysis and subgroup results of Sumac supplementation on FBG*							
Overall effect	9	499	−0.29 (−0.50, −0.07)	0.01	0.16	31.9	
Health status							
Endocrine/metabolic conditions	6	348	−0.27 (−0.56, 0.00)	0.052	0.125	42.0	0.953
Other Conditions	3	151	−0.29 (−0.69, 0.10)	0.153	0.210	35.9	
Duration of intervention (weeks)							
6 < weeks	301	5	−0.44 (−0.73, −0.16)	**0.002**	0.176	36.8	0.053
6 ≥ weeks	198	4	−0.04 (−0.33, 0.23)	0.740	0.831	0	
Sumac dosage (g/day)							
≥ 3 g	276	5	−0.26 (−0.52, −0.006)	0.045	0.328	13.6	0.774
< 3 g	223	4	−0.29 (−0.70, 0.11)	0.163	0.073	56.9	
*Analysis and subgroup results of Sumac supplementation on HOMA‐IR*							
Overall effect	6	319	−0.68 (−1.28, −0.09)	**0.024**	< 0.001	84.5	
Health status							
Endocrine/metabolic conditions	3	168	−0.80 (−2.02, 0.42)	0.200	< 0.001	92.6	0.724
Other conditions	3	151	−0.56 (−1.02, −0.11)	**0.015**	0.147	47.8	
Duration of intervention (weeks)							
6 < weeks	3	178	−1.17 (−1.81, −0.53)	**< 0.001**	0.021	74.1	0.027
6 ≥ weeks	3	141	−0.19 (−0.78, 0.39)	0.519	0.046	67.4	
Sumac dosage (g/day)							
≥ 3 g	3	143	−0.59 (−1.08, −0.09)	**0.019**	0.119	52.9	0.795
< 3 g	3	176	−0.76 (−1.94, 0.42)	0.206	< 0.001	92.6	
*Analysis and subgroup results of Sumac supplementation on serum insulin*							
Overall effect	8	452	−0.48 (−0.90, −0.07)	0.023	0.00	78.7	
Health status							
Endocrine/metabolic conditions	5	301	−0.39 (−1.02, 0.23)	0.215	0.00	85.8	0.534
Other Conditions	3	151	−0.63 (−1.06, −0.21)	0.003	0.189	40	
Duration of intervention (weeks)							
6 < weeks	5	311	−0.64 (−1.21, −0.07)	0.026	0.00	83	0.279
6 ≥ weeks	3	141	−0.20 (−0.76, 0.35)	0.470	0.063	63.9	
Sumac dosage (g/day)							
≥ 3 g	5	276	−0.38 (−0.75, −0.009)	0.045	0.049	58	0.665
< 3 g	3	176	−0.62 (−1.62, 0.38)	0.227	< 0.001	90.2	
*Analysis results of Sumac supplementation on hs‐CRP*							
Overall effect	5	288	−0.36 (−0.77, 0.06)	0.092	0.017	66.9	
Health status							
Endocrine/metabolic conditions	4	243	−0.35 (−0.88, 0.16)	0.182	0.007	75.1	0.97
Other Conditions	1	45	−0.34 (−0.93, 0.24)	0.252	0.017	66.9	
Duration of intervention (weeks)							
6 < weeks	3	196	−0.41 (−1.11, 0.28)	0.24	0.003	82.4	0.68
6 ≥ weeks	2	92	−0.24 (−0.65, 0.16)	0.23	0.655	0	
Sumac dosage (g/day)							
≥ 3 g	3	161	−0.13 (−0.44, 0.17)	0.390	0.66	0	0.30
< 3 g	2	127	−0.62 (−1.51, 0.26)	0.168	0.016	82.8	
*Analysis and subgroup results of Sumac supplementation on HDL*							
Overall effect	8	481	0.40 (0.10, 0.70)	0.008	0.012	60.9	
Health status							
Endocrine/metabolic conditions	4	262	0.25 (−0.10, 0.60)	0.165	0.10	51.1	0.30
Other Conditions	4	219	0.57 (0.07, 1.00)	**0.025**	0.02	68.5	
Duration of intervention (weeks)							
6 < weeks	4	259	0.64 (0.13, 1.1)	**0** **.013**	0.010	73.5	0.112
6 ≥ weeks	4	222	0.18 (−0.08, 0.4)	0.177	0.552	0	
Sumac dosage (g/day)							
≥ 3 g	3	172	0.18 (−0.15, 0.51)	0.287	0.301	15.4	0.209
< 3 g	5	309	0.52 (0.10, 0.93)	0.013	0.013	68.4	
*Analysis and subgroup results of Sumac supplementation on LDL*							
Overall effect	8	481	−0.37 (−0.66, −0.08)	0.011	0.019	58.2	
Health status							
Endocrine/metabolic conditions	4	262	−0.52 (−1.02, −0.03)	**0.035**	0.009	73.8	0.226
Other Conditions	4	219	−0.18 (−0.45, 0.09)	0.188	0.605	0	
Duration of intervention (weeks)							
6 < weeks	4	259	−0.44 (−0.99, 0.10)	0.111	0.004	77.7	0.567
6 ≥ weeks	4	222	−0.26 (−0.53, 0.00)	0.048	0.549	0	
Sumac dosage (g/day)							
≥ 3 g	3	172	−0.27 (−0.58, 0.02)	0.073	0.688	0	0.637
< 3 g	5	309	−0.41 (−0.86, 0.04)	0.076	0.004	74.1	
*Analysis and subgroup results of Sumac supplementation on TC*							
Overall effect	8	481	−0.41 (−0.71, −0.11)	0.007	0.013	60.7	
Health status							
Endocrine/metabolic conditions	4	262	−0.68 (−1.04, −0.31)	**< 0.001**	0.100	52.0	0.012
Other Conditions	4	219	−0.09 (−0.37, 0.17)	0.475	0.629	0	
Duration of intervention (weeks)							
6 < weeks	4	259	−0.49 (−0.99, 0.01)	0.057	0.010	73.8	0.560
6 ≥ weeks	4	22	−0.31 (−0.64, 0.02)	0.072	0.019	37.1	
Sumac dosage (g/day)							
≥ 3 g	3	172	−0.31 (−0.62, −0.01)	0.042	0.658	0	0.595
< 3 g	5	309	−0.46 (−0.93, 0.00)	0.050	0.003	75.5	
*Analysis and subgroup results of Sumac supplementation on TG*							
Overall effect	7	436	−0.22 (−0.42, −0.03)	0.021	0.611	0	
Health status							
Endocrine/metabolic conditions	4	262	−0.36 (−0.61, −0.12)	0.004	0.839	0	0.076
Other conditions	3	174	−0.01 (−0.31, 0.3)	0.962	0.785	0	
Duration of intervention (weeks)							
6 < weeks	4	259	−0.31 (−0.56, −0.06)	**0.017**	0.546	0	0.324
6 ≥ weeks	3	177	−0.11 (−0.41, 0.18)	0.461	0.499	0	
Sumac dosage (g/day)							
≥ 3 g	2	127	−0.28 (−0.64 0.08)	0.126	0.595	0	0.725
< 3 g	5	3	−0.20 (−0.43, 0.03)	0.083	0.395	2.1	

*Note:* Values shown in bold indicate statistically significant associations for the corresponding variable.

Abbreviations: BMI, body mass index; FBG, fasting blood glucose; HDL, high‐density lipoprotein; HOMA‐IR, homeostatic model assessment of insulin resistance; hs‐CRP, high‐sensitivity C‐reactive protein; LDL, low‐density lipoprotein; TC, total cholesterol; TG, triglycerides; WC, waist circumference; WHR, waist‐to‐hip ratio.

Regarding glycemic parameters, health status, duration and dosage of Sumac supplementation can be sources of heterogeneity in FBG, HOMA‐IR, and insulin. Sumac supplementation for more than 6 weeks with a dosage of 3 g/day or more in patients with other conditions can significantly decrease FBG, HOMA‐IR, and insulin. However, in other subgroups Sumac supplementation did not change glycemic parameters in comparison with the control group.

In terms of lipid profile, Sumac supplementation for more than 6 weeks and a dosage of less than 3 g/day in other conditions significantly improves HDL levels in patients. Sumac supplementation decreases LDL, TC and TG levels significantly in patients with endocrine/metabolic conditions, and a dosage of ≥ 3 g for TC and a duration of ≥ 6 weeks for TG had similar decreasing effects in comparison with the placebo group.

### 3.9 Meta‐Regression and Non‐Linear Dose–Response Analysis

Figures S1–S4 showcase a comprehensive meta‐regression analysis that rigorously assessed the impact of Sumac doses and intervention duration on cardiovascular risk variables. Additionally, using a non‐linear dose–response regression model, our aim was to unravel the intricate relationship between Sumac supplementation and cardiovascular outcomes, and results of the dose–response analysis indicated that the optimum dosage of Sumac consumption is 2 g/day, and it has the most improving effect on HDL (*p* < 0.001) and a decreasing effect on CRP level (*p* = 0.04).

### 3.10 GRADE Assessment

The GRADE profile of Sumac supplementation on the outcomes with the certainty in outcomes is shown in Table 4.

**TABLE 4:** Grade profile of Sumac supplementation on cardiovascular risk factors.

Outcomes	Risk of bias	Inconsistency	Indirectness	Imprecision	Publication Bias	Number (INT/CON)	SMD (95% CI)	Quality of evidence
BMI	Serious^a^	Not serious	Serious^b^	Serious^c^	None	341/302	−0.14 (−0.30, 0.02)	⨁◯◯◯ Very low
WC	Not serious	Very serious^d^	Serious^b^	Serious^c^	None	233/226	−0.10 (−0.28, 0.08)	⨁◯◯◯ Very low
Weight	Serious^a^	Very serious^d^	Serious^b^	Serious^c^	None	273/266	−0.12 (−0.29, 0.05)	⨁◯◯◯ Very low
WHR	Not serious	Not serious	Serious^b^	Very serious^c^	None	227/219	−0.13 (−0.33 0.07)	⨁◯◯◯ Very low
FBG	Serious^e^	Not serious	Serious^b^	Not serious	None	254/245	−0.29 (−0.50, −0.07)	⨁⨁◯◯ Low
HOMA‐IR	Not serious	Very serious^d^	Serious^b^	Serious^c^	None	162/157	−0.68 (−1.28, −0.09)	⨁◯◯◯ Very low
Serum Insulin	Serious^e^	Very serious^d^	Serious^b^	Not serious	None	231/221	−0.48(−0.90, −0.07)	⨁◯◯◯ Very low
Hs‐CRP	Not serious	Very serious^d^	Serious^b^	Extremely serious^c^	None	148/140	−0.36 (−0.77, 0.06)	⨁◯◯◯ Very low
HDL	Not serious	Serious^d^	Serious^b^	Not serious	None	257/224	0.40 (0.10, 0.70)	⨁⨁◯◯ Low
LDL	Not serious	Serious^d^	Serious^b^	Not serious	None	257/224	−0.37 (−0.66, −0.08)	⨁⨁◯◯ Low
TC	Not serious	Serious^d^	Serious^b^	Not serious	None	257/224	−0.41 (−0.75, −0.08)	⨁⨁◯◯ Low
TG	Not serious	Not serious	Serious^b^	Not serious	None	257/224	−0.41 (−0.71, −0.11)	⨁⨁⨁◯ Moderate

Abbreviations*:* BMI, Body Mass Index; CI, Confidence Interval; CON, Control Group; DBP, Diastolic Blood Pressure; FBG, Fasting Blood Glucose; HbA1c, Hemoglobin A1C; HDL, High‐Density Lipoprotein; HOMA‐IR, Homeostatic Model Assessment of Insulin Resistance; hs‐CRP, High‐Sensitivity C‐Reactive Protein; INT, Intervention Group; LDL, Low‐Density Lipoprotein; MDA, Malondialdehyde; SBP, Systolic Blood Pressure; SMD, Standardised Mean Difference; TC, Total Cholesterol; TG, Triglycerides; WC, Waist Circumference; WHR, Waist‐to‐Hip Ratio.

^a^ More than 20% of RCTs for this outcome had a high risk of bias for at least one component of the Cochrane risk of bias tool. Those biases had a significant effect on the results of RCTs.

^b^ Downgraded for indirectness in country.

^c^ Downgraded since the 95% CI crosses the threshold of interest.

^d^ The I^2^ value was > 50% (or Heterogeneity among the studies was high).

^e^ Downgraded since more than 50% of the participants were from high risk of bias studies.

#### 3.10.1 BMI

The evidence quality for BMI was rated as “very low” due to multiple downgrades. There was a serious risk of bias, as more than 20% of the RCTs measuring this outcome had a high risk of bias in at least one component of the Cochrane risk of bias tool, significantly affecting results. Indirectness was serious due to country‐specific limitations, suggesting potential issues with generalising findings to broader populations. Imprecision was serious because the 95% confidence interval (−0.30, 0.02) crossed the threshold of interest, indicating uncertainty in the true effect. While no publication bias was detected, the cumulative impact of these limitations severely diminished confidence in the reported effect size (SMD −0.14), resulting in very‐low‐quality evidence despite the relatively large sample size (341 intervention/302 control).

#### 3.10.2 WC

Waist circumference was assessed as “very‐low‐quality” quality evidence despite not having serious risk of bias. The major limitations included very serious inconsistency (*I* ^2^ value > 50%), indicating substantial heterogeneity among studies that couldn't be explained by chance alone. Serious indirectness due to country limitations affected generalizability, while serious imprecision was noted as the confidence interval (−0.28, 0.08) crossed the threshold of interest, including both potential benefit and harm. Although publication bias was not detected, the combination of these significant methodological concerns in the 233 intervention and 226 control participants substantially undermined confidence in the small observed effect (SMD −0.10), resulting in very‐low‐quality evidence.

#### 3.10.3 Weight

The evidence for weight outcomes was rated “very‐low‐quality” due to multiple serious limitations. There was a serious risk of bias, with more than 20% of RCTs having a high risk in at least one Cochrane risk assessment component that significantly affected results. Very serious inconsistency was evident with an *I* ^2^ value exceeding 50%, demonstrating substantial unexplained heterogeneity across studies. Serious indirectness related to country limitations restricted generalizability, while serious imprecision was noted as the confidence interval (−0.29, 0.05) crossed the threshold of interest, including both potential benefit and no effect. Although publication bias wasn't detected, these cumulative limitations severely undermined confidence in the observed small effect size (SMD −0.12) across the 273 intervention and 266 control participants.

#### 3.10.4 WHR

Waist‐to‐hip ratio evidence was graded as “very‐low‐quality” despite having no serious risk of bias or inconsistency issues. The downgrade was primarily due to serious indirectness related to country limitations affecting generalizability, and very serious imprecision with the confidence interval (−0.33, 0.07) showing substantial uncertainty by crossing the threshold of interest. This very serious imprecision suggests that the true effect could range from moderate benefit to slight harm. With no publication bias detected, the evidence quality still suffered substantially due to these limitations, particularly imprecision, resulting in very low confidence in the observed effect size (SMD −0.13) across 227 intervention/219 control participants.

#### 3.10.5 FBG

Fasting blood glucose evidence was rated as “low‐ quality”, representing somewhat higher confidence than most outcomes in this study. It was downgraded due to a serious risk of bias, as more than 50% of participants came from studies with a high risk of bias. Serious indirectness was also noted due to country limitations affecting generalizability. However, no serious inconsistency was detected (*I* ^2^ value < 50%), and imprecision was not serious as the confidence interval (−0.50, −0.07) did not cross the threshold of interest, suggesting a consistent small‐to‐moderate beneficial effect. No publication bias was detected. The resulting “low‐quality” rating for this effect (SMD −0.29) across 254 intervention/245 control participants indicates limited confidence in the estimate, but represents higher certainty than “very‐low‐quality” outcomes.

#### 3.10.6 HOMA‐IR

HOMA‐IR evidence was assessed as “very‐low‐quality” despite having no serious risk of bias. The major concerns included very serious inconsistency with *I* ^2^ values exceeding 50%, indicating substantial unexplained heterogeneity between studies. Serious indirectness due to country limitations affected the generalizability of the findings. Serious imprecision was evident as the confidence interval (−1.28, −0.09) crossed the threshold of interest, showing substantial uncertainty despite a statistically significant effect. While no publication bias was detected, these significant methodological limitations substantially undermined confidence in the moderate‐to‐large observed effect size (SMD −0.68) across the 162 intervention/157 control participants, resulting in “very‐low‐quality” evidence.

#### 3.10.7 Serum Insulin

The evidence quality for serum insulin was rated “very‐low” due to multiple serious limitations. There was a serious risk of bias, as more than 50% of participants came from studies with a high risk of bias. Very serious inconsistency was present with *I* ^2^ values exceeding 50%, demonstrating substantial unexplained heterogeneity. Serious indirectness was noted due to country limitations affecting generalizability. Despite these issues, imprecision was not serious as the confidence interval (0.90, −0.07) did not cross the zero‐effect threshold, suggesting a consistent benefit. No publication bias was detected. However, the cumulative impact of these limitations severely reduced confidence in the moderate observed effect (SMD −0.48) across 231 intervention/221 control participants.

#### 3.10.8 Hs‐CRP

Hs‐CRP evidence was rated as “very‐low‐quality” with multiple serious concerns despite having no serious risk of bias. It had very serious inconsistency with *I* ^2^ values exceeding 50%, indicating substantial unexplained heterogeneity. Serious indirectness due to country limitations affected generalizability. Most critically, it had extremely serious imprecision—a double downgrade—as the confidence interval (−0.77, 0.06) not only crossed the threshold of interest but was particularly wide, indicating high uncertainty in the true effect. Additionally, the small sample size (148 intervention/140 control) further contributed to imprecision concerns. Although no publication bias was detected, these significant methodological limitations resulted in “very‐low” confidence in the observed effect size (SMD −0.36).

#### 3.10.9 HDL

The evidence for HDL was rated as “low‐ quality”, representing moderate confidence compared to other outcomes in this study. No serious risk of bias was detected. However, serious inconsistency was present with *I* ^2^ values between 25% and 50%, indicating moderate heterogeneity. Serious indirectness due to country limitations affected generalizability. Imprecision was not serious as the confidence interval (0.10, 0.70) did not cross the threshold of interest, suggesting a consistent beneficial effect. No publication bias was detected. The resulting “low‐ quality” rating for this small‐to‐moderate positive effect (SMD 0.40) across 257 intervention/224 control participants indicates limited confidence in the estimate, but represents higher certainty than “very low” quality outcomes.

#### 3.10.10 LDL

LDL evidence was assessed as “low‐ quality” with some important limitations. No serious risk of bias was detected. Serious inconsistency was present with I^2^ values between 25% and 50%, indicating moderate heterogeneity between studies. Serious indirectness due to country limitations affected generalizability. Imprecision was not serious as the confidence interval (0.66, −0.08) did not cross the threshold of interest, suggesting a consistent beneficial effect in reducing LDL levels. No publication bias was detected. The resulting “low‐ quality” rating for this small‐to‐moderate effect (SMD −0.37) across 257 intervention/224 control participants indicates limited confidence in the estimate, but represents higher certainty than most outcomes in this study.

#### 3.10.11 Tc

The evidence quality for total cholesterol was rated as “low‐ quality” with similar limitations to other lipid parameters. No serious risk of bias was detected. Serious inconsistency was present with *I* ^2^ values between 25% and 50%, indicating moderate heterogeneity. Serious indirectness due to country limitations affected generalizability. Imprecision was not serious as the confidence interval (−0.75, −0.08) did not cross the threshold of interest, suggesting a consistent beneficial effect in reducing total cholesterol. No publication bias was detected. The resulting “low‐quality” rating for this moderate effect (SMD −0.41) across 257 intervention / 224 control participants indicates limited confidence in the estimate but represents better certainty than most outcomes in this review.

#### 3.10.12 TG

Triglycerides evidence was rated as “moderate‐quality”, representing the highest confidence level among all outcomes in this systematic review. No serious risk of bias was detected. Unlike other lipid parameters, no serious inconsistency was present (*I* ^2^ value < 25%), indicating good consistency across studies. There was serious indirectness due to country limitations affecting generalizability, which represented the only major limitation. Imprecision was not serious as the confidence interval (−0.71, −0.11) did not cross the threshold of interest, suggesting a consistent beneficial effect in reducing triglyceride levels. No publication bias was detected. The resulting “moderate‐quality” rating for this moderate effect size (SMD −0.41) across 257 intervention/224 control participants indicates reasonable confidence in the estimate—the highest among all outcomes assessed.

## 4 Discussion

This systematic review and dose–response meta‐analysis aims to evaluate the effects of Sumac consumption on cardiovascular outcomes in adults. We identified 15 RCTs with 917 participants. These studies suggest that Sumac supplementation had significant beneficial effects on serum cardiovascular outcomes including glycemic profile and lipid profile. Sumac consumption was not significantly associated with more cardiovascular risk factors. Importantly, beyond statistical significance, the significant changes in fasting FBG and lipids are clinically relevant. SMD reductions in FBG and atherogenic lipids (LDL‐C, TG) in our analysis correspond to modest but meaningful shifts that have been linked in cohort and trial data to lower cardiovascular event rates, particularly in high‐risk groups such as individuals with T2DM and NAFLD [45, 46, 47, 48]. For example, meta‐epidemiological models indicate that each ~1 mmol/L (≈39 mg/dL) reduction in LDL‐C is associated with roughly a 20%–25% relative risk reduction in major vascular events; while our pooled SMDs cannot be mapped one‐to‐one to mg/dL without study‐level SDs, the direction and consistency of LDL‐C lowering suggest that even small absolute reductions, when accrued alongside improvements in TG and HDL‐C, may translate into cardiovascular risk reduction [45, 46, 49]. Similarly, SMD‐based improvements in FBG and HOMA‐IR for better glycemic control and insulin sensitivity are associated with lower microvascular risk and, in T2DM, incremental macrovascular benefit when sustained [48, 50]. In NAFLD, improvements in atherogenic dyslipidemia and insulin resistance are mechanistic drivers of reduced cardiometabolic risk, supporting the plausibility that the observed biomarker changes could yield clinical benefit over time [51, 52].

Cardiovascular diseases encompass a broad spectrum of conditions related to the heart and blood vessels [1]. In recent investigations, the substantial promise of medicinal plants and their compounds has been emphasized as valuable assets for preventing and addressing a variety of health concerns [53, 54, 55]. Sumac is acknowledged as a medicinal spice with diverse properties such as numerous anti‐cardiovascular and metabolic risk factors [13, 14, 15, 16].

According to the study's findings, Sumac had a significant decrease in FBG, HOMA‐IR, Serum Insulin, LDL, TC, TG, while there was an increase in HDL compared with the placebo group. The results of the present study are in line with some clinical studies that have been done so far, which indicated that Sumac supplementation had a significant decrease in anthropometric parameters [13, 39, 56]. Numerous pathways contribute to the impact of dietary polyphenols on managing obesity; one of them involves mitigating oxidative stress [57, 58]. Oxidative stress, implicated in the onset of obesity, can promote the accumulation of white adipose tissue and influence eating habits [59]. Another potential mechanism through which polyphenol intake may influence obesity is by lowering inflammation levels [58, 60]. The subsequent mechanisms contributing to the positive effects of Sumac on obesity management are likely associated with its elevated total phenolic acid content, particularly gallic acid in Sumac [61]. Gallic acid appears to play a crucial role in modulating oxidative stress [61]. Another potential mechanism involves the potential inhibition of lipid absorption from the gastrointestinal tract by polyphenols, which can serve as natural inhibitors of pancreatic lipase and thus have a reducing effect on adiposity indices [25].

In this meta‐analysis, Sumac supplementation decreased the glycemic profile including FBG, HOMA‐IR and serum insulin. Sumac stands out as a widely embraced spice abundant in diverse bioactive phytocomponents including flavonoids and phenolic acid, which exhibit a range of biological effects, such as anti‐inflammatory and hypoglycemic activities, consequently safeguarding the functionality of beta cells in the pancreas [16, 62, 63, 64]. The insulin‐like properties exhibited by polyphenols of sumac, coupled with its antioxidant attributes, position it as a formidable candidate for the regulation of FBG levels [65]. Previously, Shidfar et al. showed that consumption of 3 g/day of sumac powder for 12 weeks could decrease the serum FBG [14]. Our findings align with the mentioned results, supporting the idea that Sumac supplementation for more than 6 weeks and at a dose of 3 g/day or more has the potential to reduce oxidative stress and consequently improve insulin resistance. Another mechanism that shows the hypoglycemic activity of Sumac is its effect on the signalling pathways of SIRT1, PI3K/Akt and AMPK, which play a role in improving glucose uptake and insulin sensitivity [62, 66]. Giancarlo and his research team illustrated that the hypoglycemic activity of Sumac extract was evidenced through the inhibition of alpha‐amylase activity [66].

Sumac supplementation results in a substantial decrease in TC, LDL‐C, and TG, as well as an increase in HDL‐C levels, according to the findings of this systematic review and meta‐analysis. A subgroup study demonstrated that endocrine and metabolic conditions could influence the effect of Sumac on blood lipids in PCOS, T2DM, Dyslipidemia, NAFLD, and MetS. The cholesterol‐lowering properties of Sumac have been attributed to flavonoids, including quercetin [67]. The polyphenolic elements found in Sumac demonstrate an effective reduction in lipid absorption from the gastrointestinal system, coupled with an augmentation in the excretion of bile acids [68]. Sumac's phenolic compounds are acknowledged for their ability to inhibit HMG‐CoA reductase. This inhibition leads to an enhancement in the expression of peroxisome proliferator‐activated receptor alpha (PPAR‐α) and the synthesis of Apo A‐1 in the liver, contributing favourably to the production of HDL‐C [69]. Moreover, experimental studies have shown that Sumac extract, along with its bioactive components like fibre, tannins phenolic acids, anthocyanins, and flavonoids, exhibits antihyperlipidemic effects and increases ApoA1 and HDL [70, 71]. To sum up, these mechanistic pathways align with the observed biomarker shifts and support clinical plausibility: in T2DM, dual improvements in glycemia and atherogenic lipids address two major, independent determinants of cardiovascular risk; in NAFLD, lipid lowering (particularly TG and LDL‐C) and improved insulin sensitivity are expected to reduce the high burden of cardiovascular morbidity that exceeds liver‐related outcomes in this population [51, 52].

### 4.1 Clinical Implications of Findings

The findings of this meta‐analysis offer valuable clinical implications for healthcare practitioners managing patients with metabolic and cardiovascular disorders. For patients with endocrine/metabolic conditions such as PCOS, T2DM, NAFLD, and MetS. Sumac supplementation demonstrates promise in improving lipid profiles, as evidenced by significant reductions in LDL, TC, and TG levels. Similarly, for patients with conditions like primary hyperlipidemia, haemodialysis, and obesity, Sumac supplementation shows beneficial effects on glycemic control metrics (FBG, HOMA‐IR) and HDL elevation. The dose–response analysis suggests that an optimal daily dose of 2 g of Sumac appears most effective in improving HDL levels and reducing CRP levels, making this a practical recommendation for clinical application. Healthcare providers should consider recommending longer supplementation periods (> 6 weeks) to achieve significant improvements in FBG, HOMA‐IR, serum insulin, HDL, and TG levels, as shorter intervention periods showed negligible effects across measured parameters.

For dietitians and nutritionists advising patients with cardiovascular risk factors, Sumac supplementation represents a potential adjunctive dietary intervention alongside standard treatment protocols. The significant improvements in glycemic control parameters (FBG, HOMA‐IR, serum insulin) and lipid profiles suggest that Sumac may be particularly beneficial for patients with insulin resistance and dyslipidemia, potentially reducing their overall cardiovascular risk burden. The moderate certainty evidence for TG reduction and low certainty evidence for HDL, LDL, and TC improvements indicate that Sumac's most reliable effects are on lipid metabolism, though improvements in other parameters should be considered promising but preliminary. Importantly, while anthropometric measurements showed no significant changes with Sumac supplementation, the spice's beneficial effects on metabolic parameters independent of weight loss suggest it may offer metabolic benefits that complement traditional weight management strategies. Clinically, this implies that patients may realize cardiometabolic benefit from Sumac even without weight loss, which is a relevant consideration for those who struggle to achieve clinically meaningful weight reduction [72, 73].

When implementing Sumac supplementation in clinical practice, healthcare professionals should consider individual patient characteristics, concurrent medications, and therapeutic goals. The current evidence suggests Sumac is most beneficial for patients with endocrine/metabolic disorders when targeting lipid profile improvements, while other conditions may benefit more from its glycemic control effects. While the GRADE assessment indicates varying levels of certainty across outcomes, with TG reduction having the highest reliability, the overall safety profile and multiple potential benefits make Sumac a reasonable consideration for appropriate patients. However, clinicians should acknowledge the geographical limitations of current evidence (all studies conducted in Iran) and the need for more diverse and extensive research before making broad generalisations. Pending additional high‐quality clinical trials, Sumac supplementation at 2–3 g daily for periods exceeding 6 weeks could be considered as part of comprehensive management plans for patients with metabolic syndrome, diabetes, dyslipidemia, and related cardiovascular risk factors.

### 4.2 Strengths and Limitations

A paramount strength of our meta‐analysis lies in its methodological rigor and adherence to established scientific standards. By strictly following PRISMA guidelines and registering our protocol with PROSPERO, we ensured transparency and reproducibility throughout the research process. Our comprehensive search strategy encompassed multiple electronic databases and manual reference screening, which minimized the risk of overlooking crucial studies and allowed us to capture the most recent evidence through March 2025. The application of the GRADE framework to assess the strength of evidence represents a significant advancement over previous reviews, offering a nuanced evaluation of the quality and consistency of included studies across different outcomes. Furthermore, our employment of sophisticated statistical techniques, including meta‐regression and non‐linear dose–response analyses, provides valuable insights into optimal dosing and treatment durations for Sumac supplementation across diverse populations and health outcomes. This robust methodological approach enhances the validity of our conclusions and contributes significantly to the scientific understanding of Sumac's therapeutic potential.

Our study offers several substantial improvements over the meta‐analysis conducted by Bahari et al. (2023) [74], which focused exclusively on Sumac's effects on lipid profiles. A critical limitation of their work was the inclusion of methodologically problematic studies, particularly the Rouhi‐Boroujeni et al. trial [75], where the intervention group received a combination of lovastatin and Sumac while the control group received only lovastatin—introducing a significant confounding variable. Additionally, we identified serious discrepancies in their participant allocation methodology, especially in the Alahnoori et al. study [41], where the placebo group was inappropriately divided into separate cohorts, artificially inflating the sample size and potentially skewing results. Their work also contained inaccuracies in reported dosages, notably misreporting the Afandak et al. [32] study's 3‐g dosage as 2 g, which led to erroneous categorization in subgroup analyses. Furthermore, their qualitative assessment was inadequate, particularly in evaluating attrition bias for studies with dropout rates exceeding Cochrane's recommended thresholds. These issues are clearly and comprehensively outlined in a recently published letter to the editor in this field [76]. Our meta‐analysis addresses these limitations through rigorous inclusion criteria, accurate data extraction, and comprehensive risk of bias assessment.

Similarly, our work substantially improves upon Jafarpour et al.'s (2024) [77] systematic review, which suffered from significant methodological flaws that undermine its conclusions. Their most notable limitation was the inclusion of studies with confounded interventions, such as the Ardalani et al. trial [61], where participants received captopril with Sumac, making it impossible to attribute observed effects solely to Sumac supplementation. Their search strategy was also inadequate, limited to only three databases and lacking a detailed search protocol, which raises concerns about the comprehensiveness of their literature review. We noted critical errors in their handling of crossover designs, particularly misreporting participant numbers in the Asgary study [20], and inappropriate statistical handling of data from studies with multiple intervention arms. Additionally, their GRADE assessment failed to acknowledge the indirectness limitation of having all included studies conducted exclusively in Iran, which affects the generalizability of findings. Our meta‐analysis overcomes these shortcomings through more stringent inclusion criteria, a more comprehensive search strategy, and proper statistical handling of complex trial designs.

The third meta‐analysis by Vajdi et al. (2024) [78] also presents several limitations that our study addresses. Most notably, their work lacks a GRADE assessment, which is essential for evaluating the certainty of evidence for different outcomes. Their analysis is further limited by the absence of dose–response investigations, which are crucial for establishing optimal therapeutic dosages. Their search was conducted only until December 2023, missing more recent evidence that could significantly impact conclusions given the limited number of trials in this field. Our meta‐analysis extends beyond their work by incorporating non‐linear dose–response modelling that identified the optimal dosage of Sumac consumption (2 g/day) for maximising HDL improvement and CRP reduction. Additionally, their methodology lacked the advanced statistical techniques necessary to account for baseline differences across studies and failed to provide actionable insights regarding the mechanisms underlying Sumac's beneficial effects. Their subgroup analyses were also less comprehensive, failing to adequately examine the differential effects of Sumac across various clinical populations and intervention durations.

Despite the strengths of our meta‐analysis, several limitations must be acknowledged. The relatively small number of included studies (15 RCTs) and the moderate sample sizes introduce a degree of heterogeneity that could influence the overall results. Even with the use of random‐effects models to address this variability, the possibility of remaining confounding factors cannot be completely eliminated. Another limitation is that all included trials were conducted in Iran, which may limit the generalizability of our findings to other populations with different genetic backgrounds, dietary patterns, and environmental factors. This geographical limitation reflects the current state of the evidence base rather than a methodological choice, highlighting a significant gap in global research on Sumac supplementation that future studies should address.

Furthermore, the variability in baseline health status among participants in different studies introduces complexity in interpreting results across conditions. Our subgroup analyses attempted to address this by stratifying results based on health conditions, but larger sample sizes within each health category would provide more definitive conclusions. Additionally, the included studies utilised different forms of Sumac (varying preparations and parts of the plant), which could contribute to heterogeneity in the observed effects. The intervention durations were relatively short (4–12 weeks), which limits our ability to assess the long‐term effects and safety profile of Sumac supplementation. Finally, despite our comprehensive approach, the certainty of evidence for several outcomes was rated as “low‐quality” or “very‐low‐quality” according to the GRADE assessment, indicating that future high‐quality research is needed to strengthen the evidence base for Sumac's cardiometabolic effects. The comparison of the current study and previous meta‐analysis is provided in Table 5.

**TABLE 5:** methodological aspects of the current meta‐analysis and comparison with prior relevant reviews.

Study	Primary focus	Databases	Search end date	Risk of bias	GRADE	Included studies (*n* = participants)	Data handling issues and limitations
Current Study	Cardiometabolic factors: TC, HDL, LDL, TG, BMI, WC, weight, WHR, FBG, HOMA_IR, Insulin, Hs‐CRP	PubMed, Web of Science, EMBASE, Scopus, and CENTRAL	March 2025	2 studies had pure quality, 4 studies were fair and 9 studies had good quality	Yes, for all outcomes	15 (*n* = 917)	Limited generalizability, Variability in participants' baseline health status, Heterogeneity in Sumac preparations and plant parts used, short intervention durations (4–12 weeks), Low to very‐low certainty of evidence (GRADE)
Bahari et al. (2023) [62]	Lipid profile: TC, TG, LDL, HDL	PubMed, Scopus, and Web of Science	March 2023	General risk of bias for all studies was low except for one study was high.	No	7 (*n* = 530)	Included methodologically flawed trials, Participant allocation errors, Dosage inaccuracies, Inadequate quality assessment
Jafarpour et al. (2024) [68]	Cardiometabolic factors:	PubMed, Scopus, and Web of Science	August 2023	12 studies had high risk of bias and 4 studies had some concerns of bias	Yes, but failed to address indirectness (Iran‐only studies)	16 (*n* = 1225)	Inclusion of trials with confounded interventions, Inadequate search strategy, Misreported participant numbers in crossover designs, Improper statistical handling of multi‐arm trials, GRADE assessment overlooked indirectness
Vajdi et al. (2024) [70]	Cardiovascular risk factors: FBG, HbA1c, Insulin, HOMA‐IR, TG, TC, LDL, HDL, BMI, weight, WC, HC, WHR, SBP, DBP	PubMed, Embase, Scopus, Cochrane Library, Web of Science	December 2023	Not detailed	No	16 (*n* = 1185)	No GRADE assessment of evidence certainty, Lacked dose–response analysis, Search limited to December 2023 (missed newer trials), Did not adjust for baseline differences across studies, Subgroup analyses were limited and incomplete

Abbreviations: BMI, body mass index; CENTRAL, cochrane central register of controlled trials; DBP, diastolic blood pressure; EMBASE, excerpta medica database; FBG, fasting blood glucose; GRADE, grading of recommendations assessment, development and evaluation; HbA1c, Haemoglobin A1c; HC, hip circumference; HDL, high‐density lipoprotein; HOMA‐IR, homeostatic model assessment for insulin resistance; Hs‐CRP, high‐sensitivity C‐reactive protein; LDL, low‐density lipoprotein; SBP, systolic blood pressure; TC, total cholesterol; TG, triglycerides; WC, waist circumference; WHR, waist‐to‐hip ratio.

### 4.3 Future Research Directions

Building on the promising findings of our meta‐analysis and acknowledging the existing gaps in the current body of literature, future research should focus on several key areas to further elucidate the therapeutic potential of Sumac supplementation. First, there is a distinct need for long‐term, multicenter randomised controlled trials that extend well beyond the 12‐week intervention period reported in the existing studies. Extended duration trials are essential to evaluate the sustainability of Sumac's beneficial effects on glycemic control, lipid profiles, and other metabolic outcomes, as well as identifying its long‐term safety profile. Notably, because all available evidence to date originates from studies conducted in Iran, future trials should endeavour to include diverse populations with varying genetic backgrounds, dietary habits, and environmental exposures. This broader approach would enhance the external validity of the findings and ensure that clinical recommendations can be confidently generalised across different demographic groups.

In parallel with these clinical trials, mechanistic studies using advanced molecular biology and omics technologies are imperative to unravel the complex biochemical pathways through which Sumac exerts its effects. Research should target the identification of specific bioactive compounds and their molecular targets relevant to glucose metabolism, lipid homeostasis, and systemic inflammation. Detailed investigations into the modulation of regulatory hormones like leptin and the potential impact on appetite regulation could provide mechanistic insights that bridge the gap between observed clinical outcomes and underlying biological processes. Additionally, exploring how Sumac influences gut microbiota composition may reveal novel pathways contributing to its metabolic and cardioprotective effects.

Future studies should also consider a thorough dose–response analysis to determine the optimal dosing strategies tailored for various patient populations. Given our dose–response findings suggesting that a daily intake of approximately 2–3 g may be optimal for enhancing HDL levels and reducing inflammatory markers, research should further assess whether different formulations (e.g., whole fruit versus standardised extracts) produce comparable or even superior outcomes. Investigations into potential synergistic effects when Sumac is combined with conventional medications, or other natural compounds, should also be prioritised. These studies could illuminate combination strategies that maximise therapeutic benefits while mitigating potential adverse interactions, especially in patients with comorbid conditions such as metabolic syndrome, type 2 diabetes, or dyslipidemia.

**FIGURE 6:**
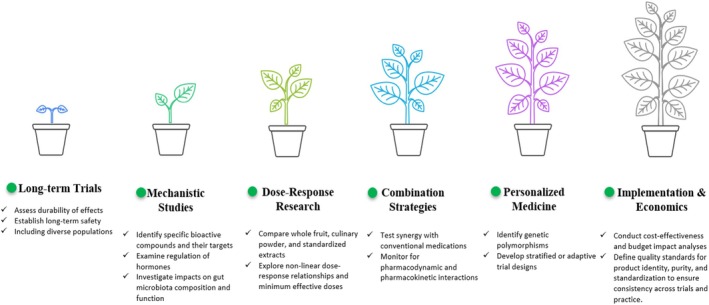
Future research directions.

Lastly, future research endeavours should integrate personalised medicine approaches by exploring genetic polymorphisms and other biomarkers that predict individual responsiveness to Sumac supplementation. Tailoring treatment based on such factors could lead to more nuanced, patient‐specific recommendations and a deeper understanding of inter‐individual variability in therapeutic outcomes. Additionally, incorporating cost‐effectiveness analyses into future studies would provide essential data for healthcare policy makers and inform clinical decision‐making regarding the incorporation of Sumac into standard treatment protocols. Together, these research directions will not only validate and extend the current findings but also pave the way for innovative, evidence‐based strategies for managing metabolic and cardiovascular health through Sumac supplementation (Figure 6).

## 5 Conclusions

The systematic review and meta‐analysis of 15 studies suggest that sumac supplementation exerts beneficial effects on glycemic control and lipid profiles in adults. However, further long‐term, well‐designed randomised controlled trials of higher quality conducted in diverse populations are needed to confirm and expand upon these findings.

## Author Contributions

Conceptualization: A.J.; Data curation: A.J., B.P.N., M.A., M.H., N.R., A.S.‐B., S.B.; Formal analysis: A.J., G.R., M.C.; Investigation: A.J., B.P.N., M.A., M.H., N.R. and A.S.‐B.; Methodology: A.J., G.R., S.B., M.C., A.A.; Project administration: A.J., A.A.; Supervision: A.J.; Validation: A.J., G.R., S.B.; Writing – original draft: A.J., N.R.; Writing – review and editing: A.J., B.P.N., M.A., M.H., A.S.‐B., G.R., S.B., M.C., A.A.

## Ethics Statement

This research followed the principles outlined in the Declaration of Helsinki. Ethical approval for the research was obtained from the Ethics Committee of Golestan University of Medical Sciences (IR.GOUMS.REC.1402.346).

## Consent

The authors have nothing to report.

## Conflicts of Interest

Ali Jafari holds an editorial position at *Systematic Reviews*. All other authors of this publication declare that they have no affiliations with, or involvement in, any organisation or entity with a financial interest (including honoraria, educational grants, participation in speakers' bureaus, memberships, employment, consultancies, stock ownership or other equity interests, expert testimony, or patent‐licensing arrangements) or a non‐financial interest (such as personal or professional relationships, affiliations, knowledge, or beliefs) relevant to the subject matter or materials discussed in this manuscript.

## Supporting information


**Table S1:** Search strategy to find potential eligible randomised controlled trials (March 2025).
**Table S2:** A summary of excluded articles after full text review.
**Figure S1:** Random‐effects meta‐regression plots of the association between sumac dosage (g/d) and cardiovascular disease outcomes (a: BMI, b: WC, C: Weight, d: WHR, e: FBG, f: HOMA‐IR, g: Serum Insulin, h: HDL, i: LDL, j: TC, k:TG, m: hsCRP).
**Figure S4:** Forest plot of the effects of chia product supplement on glycemic indices.
**Figure S2:** Forest plot detailing weighted mean difference and 95% confidence intervals (CIs) for the effect of folic acid supplementation on; (A) SBP; and (B) DBP.
**Figure S3:** Forest plot of the effects of chia product supplement on anthropometric measures.
**Figure S4:** Forest plot of the effects of chia product supplement on glycemic indices.

## Data Availability

The data that support the findings of this study are available in the Supporting Information of this article.
