# Supplementary material for: Sumac (Rhus coriaria L.) and Human Metabolic Health: A Systematic Review and Meta‐Analysis
Source: Endocrinol Diabetes Metab. 2025 Dec 2;9(1):e70135. doi: 10.1002/edm2.70135 (PMC12671535; doi:10.1002/edm2.70135)
Supplement: Supplementary file 1 — Table S1: Search strategy to find potential eligible randomised controlled trials (March 2025). Table S2: A summary of excluded articles after full text review. Figure S1: Random‐effects meta‐regression plots of the association between sumac dosage (g/d) and cardiovascular disease outcomes (a: BMI, b: WC, C: Weight, d: WHR, e: FBG, f: HOMA‐IR, g: Serum Insulin, h: HDL, i: LDL, j: TC, k:TG, m: hsCRP). Figure S4: Forest plot of the effects of chia product supplement on glycemic indices. Figure S2: Forest plot detailing weighted mean difference and 95% confidence intervals (CIs) for the effect of folic acid supplementation on; (A) SBP; and (B) DBP. Figure S3: Forest plot of the effects of chia product supplement on anthropometric measures. Figure S4: Forest plot of the effects of chia product supplement on glycemic indices. [file EDM2-9-e70135-s001.docx]

**Sumac (*Rhus coriaria L.*) and Human Metabolic Health: A Systematic Review and Meta-Analysis**

**Contents**

[**Supplementary Table 1.** Search strategy to find potential eligible randomised controlled trials (March 2025) 2](#_Toc195694457)

[**Supplementary Table 2.** A summary of excluded articles after full text review   5](#_Toc195694458)

[**Supplementary Figure 1.** Random-effects meta-regression plots of the association between sumac dosage (g/d) and cardiovascular disease outcomes 6](#_Toc195694459)

[**Supplementary Figure 2.** Random-effects meta-regression plots of the association between duration of intervention and cardiovascular disease outcomes (a: BMI, b: WC, C: Weight, d: WHR, e: FBG, f: HOMA-IR, g: Serum Insulin, h: HDL, i: LDL, j: TC, k:TG, m: hsCRP) 7](#_Toc195694460)

[**Supplementary Figure 3.** Non-linear dose-response relations between sumac dosage (g/d) and cardiovascular disease outcomes (a: BMI, b: WC, C: Weight, d: WHR, e: FBG, f: HOMA-IR, g: Serum Insulin, h: HDL, i: LDL, j: TC, k:TG) 8](#_Toc195694461)

[**Supplementary Figure 4.** Non-linear dose-response relations between duration of intervention and cardiovascular disease outcomes (a: BMI, b: WC, C: Weight, d: WHR, e: FBG, f: HOMA-IR, g: Serum Insulin, h: HDL, i: LDL, j: TC, k:TG, m:hsCRP) 9](#_Toc195694462)

[**Supplemental References** 10](#_Toc195694463)

| **Supplementary Table 1.** Search strategy to find potential eligible randomised controlled trials (March 2025) | |
| --- | --- |
| **Groups** | **Descriptors** |
| Intervention | Rhus OR Sumac* OR "rhus coriaria" OR Anacardiaceae |
| Design | Intervention OR “Intervention Study” OR “Intervention Studies” OR “controlled trial” OR random* OR placebo OR “clinical trial” OR Trial OR “randomized controlled trial” OR “randomized clinical trial” OR RCT OR blinded OR “double blind” OR “double blinded” OR trial* OR “Pragmatic Clinical Trial” OR “Cross-Over Studies” OR “Cross-Over” OR “Cross-Over Study” OR parallel OR “parallel study” OR “parallel trial” |

**PubMed**

Number of localized studies: 573

|  | **Descriptors** | **Number of studies reached** |
| --- | --- | --- |
| #1 | (Rhus[Title/Abstract] OR Sumac*[Title/Abstract] OR "rhus coriaria"[Title/Abstract] OR Anacardiaceae[Title/Abstract] OR "Rhus"[Mesh] OR "Anacardiaceae"[Mesh]) | 6,063 |
| #2 | Intervention[Title/Abstract] OR "Intervention Study"[Title/Abstract] OR "Intervention Studies"[Title/Abstract] OR "controlled trial"[Title/Abstract] OR random*[Title/Abstract] OR placebo[Title/Abstract] OR "clinical trial"[Title/Abstract] OR Trial[Title/Abstract] OR "randomized controlled trial"[Title/Abstract] OR "randomized clinical trial"[Title/Abstract] OR RCT[Title/Abstract] OR blinded[Title/Abstract] OR "double blind"[Title/Abstract] OR "double blinded"[Title/Abstract] OR trial*[Title/Abstract] OR "Pragmatic Clinical Trial"[Title/Abstract] OR "Cross-Over Studies"[Title/Abstract] OR "Cross-Over"[Title/Abstract] OR "Cross-Over Study"[Title/Abstract] OR parallel[Title/Abstract] OR "parallel study"[Title/Abstract] OR "parallel trial"[Title/Abstract] OR "Clinical Trial" [Publication Type] OR "Randomized Controlled Trial" [Publication Type] OR "Controlled Clinical Trial" [Publication Type] OR "Random Allocation"[Mesh] OR "Randomized Controlled Trials as Topic"[Mesh] OR "Pragmatic Clinical Trial" [Publication Type] OR "Pragmatic Clinical Trials as Topic"[Mesh] OR "Double-Blind Method"[Mesh] OR "Single-Blind Method"[Mesh] OR "Cross-Over Studies"[Mesh] | 3,846,152 |
| #3 | #1 AND #2 | 573 |

**Web of Science**

Number of localized studies: 356

|  | **Descriptors** | **Number of studies reached** |
| --- | --- | --- |
| #1 | TS=(Rhus OR Sumac* OR "rhus coriaria" OR Anacardiaceae ) | 5,788 |
| #2 | TS=(Intervention OR “Intervention Study” OR “Intervention Studies” OR “controlled trial” OR random* OR placebo OR “clinical trial” OR Trial OR “randomized controlled trial” OR “randomized clinical trial” OR RCT OR blinded OR “double blind” OR “double blinded” OR trial* OR “Pragmatic Clinical Trial” OR “Cross-Over Studies” OR “Cross-Over” OR “Cross-Over Study” OR parallel OR “parallel study” OR “parallel trial”) | 6,750,985 |
| #3 | #1 AND #2 | 356 |

**Scopus**

Number of localized studies: 544

|  | **Descriptors** | **Number of studies reached** |
| --- | --- | --- |
| #1 | TITLE-ABS-KEY ( rhus OR sumac* OR "rhus coriaria" OR anacardiaceae ) | 7,497 |
| #2 | TITLE-ABS-KEY ( intervention OR "Intervention Study" OR "Intervention Studies" OR "controlled trial" OR random* OR placebo OR "clinical trial" OR trial OR "randomized controlled trial" OR "randomized clinical trial" OR rct OR blinded OR "double blind" OR "double blinded" OR trial* OR "Pragmatic Clinical Trial" OR "Cross-Over Studies" OR "Cross-Over" OR "Cross-Over Study" OR parallel OR "parallel study" OR "parallel trial" ) | 8,338,298 |
| #3 | #1 AND #2 | 544 |

**Embase**

Number of localized studies: 4,598

|  | **Descriptors** | **Number of studies reached** |
| --- | --- | --- |
| #1 | 'Rhus'/exp OR 'Anacardiaceae'/exp | 12,439 |
| #2 | 'randomized controlled trial'/exp OR 'randomized controlled trial (topic)'/exp OR 'pragmatic trial'/exp OR 'clinical trial'/exp OR 'clinical trial (topic)'/exp OR 'intervention study'/exp OR 'controlled study'/exp OR 'controlled clinical trial'/exp OR 'double blind procedure'/exp OR 'single blind procedure'/exp OR 'crossover procedure'/exp OR 'parallel design'/exp | 12,321,663 |
| #3 | #1 AND #2 | 4,598 |

**Cochrane**

Number of localized studies: 144

Limits: TRIALS

|  | **Descriptors** | **Number of studies reached** |
| --- | --- | --- |
| #1 | (rhus OR sumac* OR "rhus coriaria" OR anacardiaceae):ti,ab,kw | 144 |

| **Supplementary Table 2.** A summary of excluded articles after full text review | |
| --- | --- |
| **Author, Year (Ref.)** | **Reason** |
| Sabzghabaee, 2014 (1) | Not interested population (adolescents) |
| Ardalani, 2016 (2) | Adds on design |
| Hajmohammadi, 2016 (3) | Due to insufficient or unrelated information |
| Alghadir, 2016 (4) | Due to insufficient or unrelated information |
| Rouhi-Boroujeni, 2016 (5) | Adds on design |
| Azali, 2017 (6) | Persian Language |
| Niknafs, 2021 (7) | Due to insufficient or unrelated information |
| Kwak, 2021 (8) | Due to insufficient or unrelated information |
| Amirsasan, 2024 (9) | Not interested intervention |
| Bagheri, 2024 (10) | Not interested intervention |
| Nadjarzadeh, 2024 (11) | Not interested intervention |


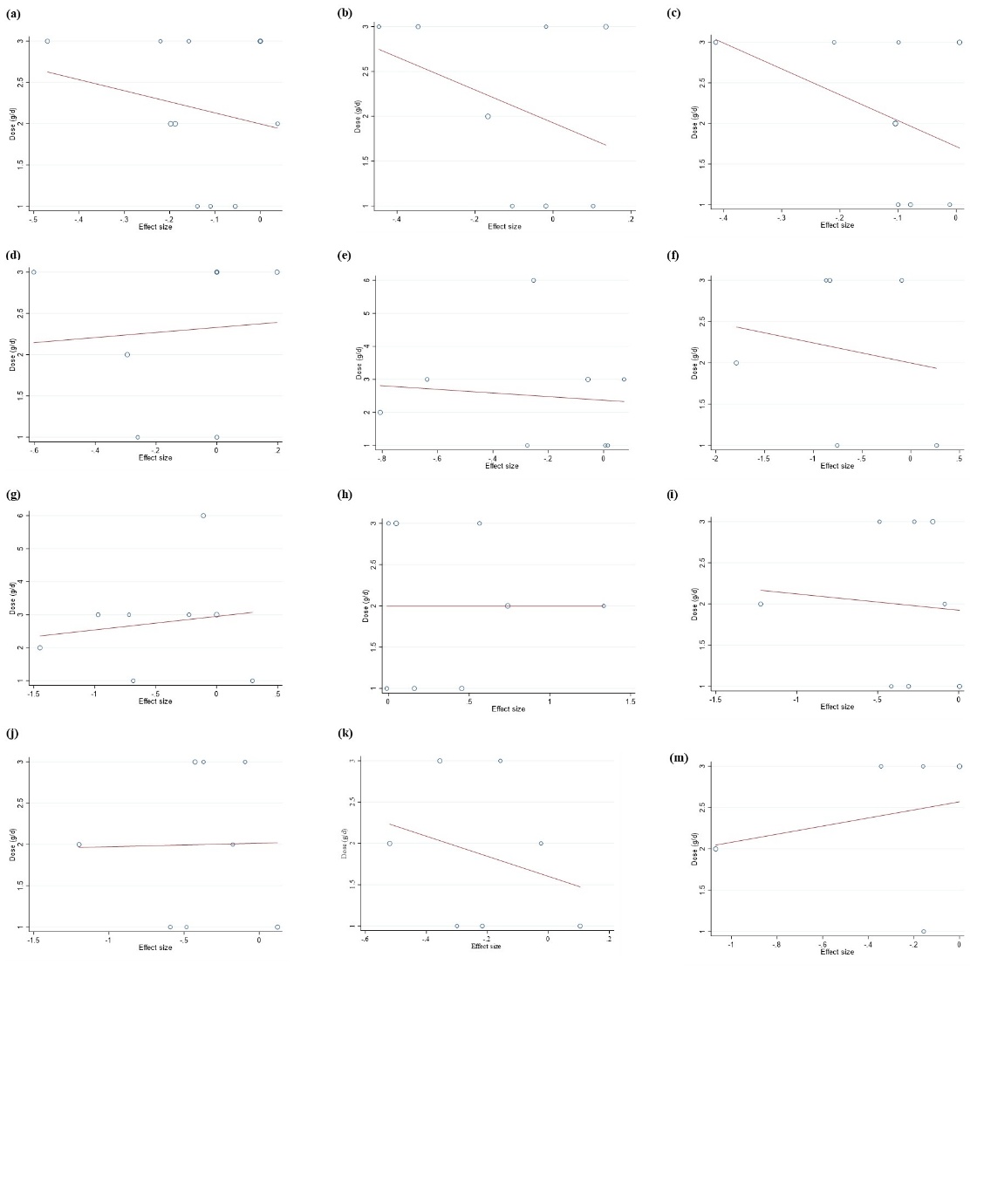


**Supplementary Figure 1.** Random-effects meta-regression plots of the association between sumac dosage (g/d) and cardiovascular disease outcomes (a: BMI, b: WC, C: Weight, d: WHR, e: FBG, f: HOMA-IR, g: Serum Insulin, h: HDL, i: LDL, j: TC, k:TG, m: hsCRP)

**Supplementary Figure 2.** Forest plot detailing weighted mean difference and 95% confidence intervals (CIs) for the effect of folic acid supplementation on; A) SBP; and B) DBP.

**Supplementary Figure 2.** Forest plot of the effects of chia product supplement on anthropometric measures.

**Supplementary Figure 4.** Forest plot of the effects of chia product supplement on glycemic indices.


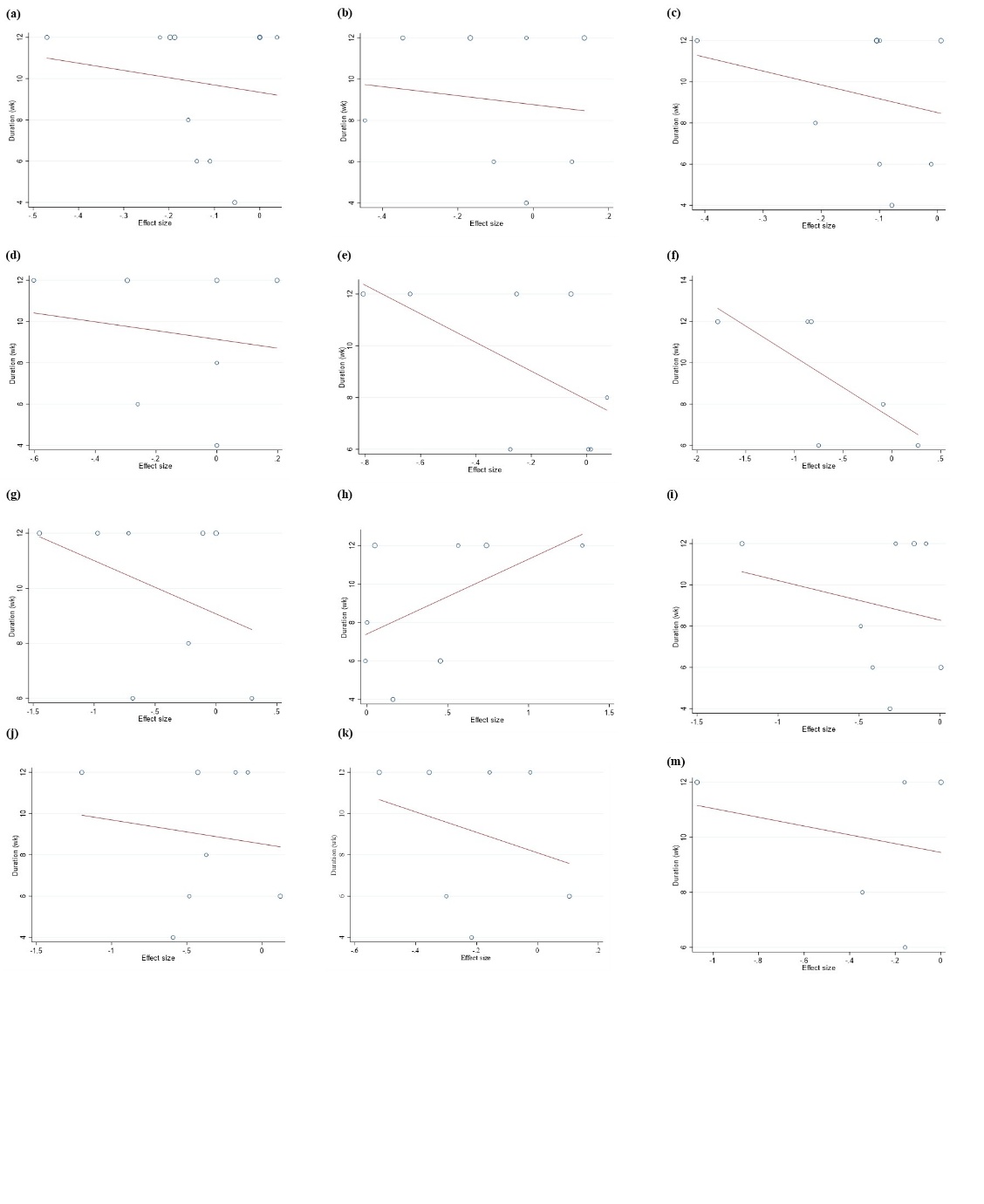


**Supplementary Figure 2.** Random-effects meta-regression plots of the association between duration of intervention and cardiovascular disease outcomes (a: BMI, b: WC, C: Weight, d: WHR, e: FBG, f: HOMA-IR, g: Serum Insulin, h: HDL, i: LDL, j: TC, k:TG, m: hsCRP)


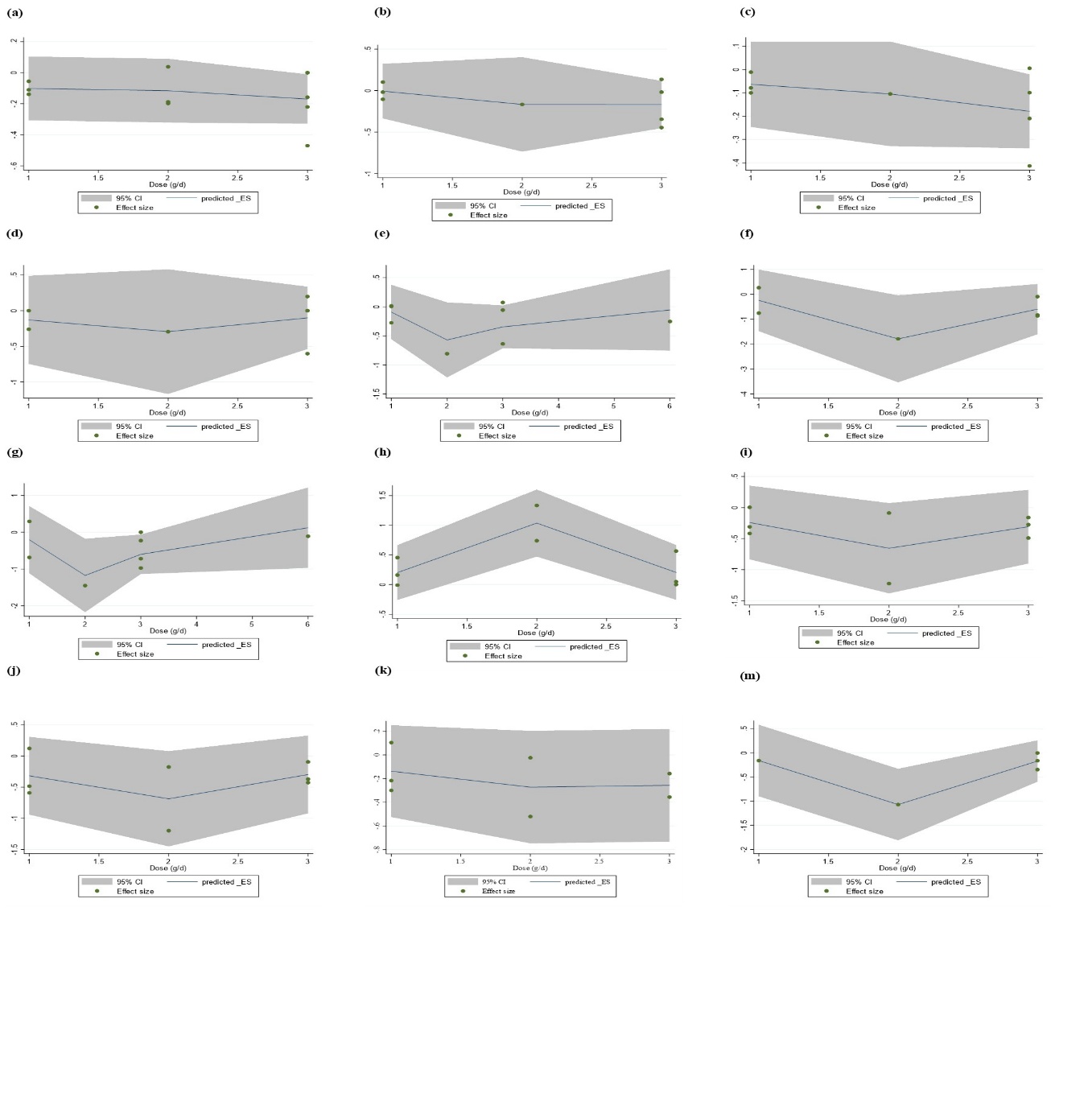


**Supplementary Figure 3.** Non-linear dose-response relations between sumac dosage (g/d) and cardiovascular disease outcomes (a: BMI, b: WC, C: Weight, d: WHR, e: FBG, f: HOMA-IR, g: Serum Insulin, h: HDL, i: LDL, j: TC, k:TG)


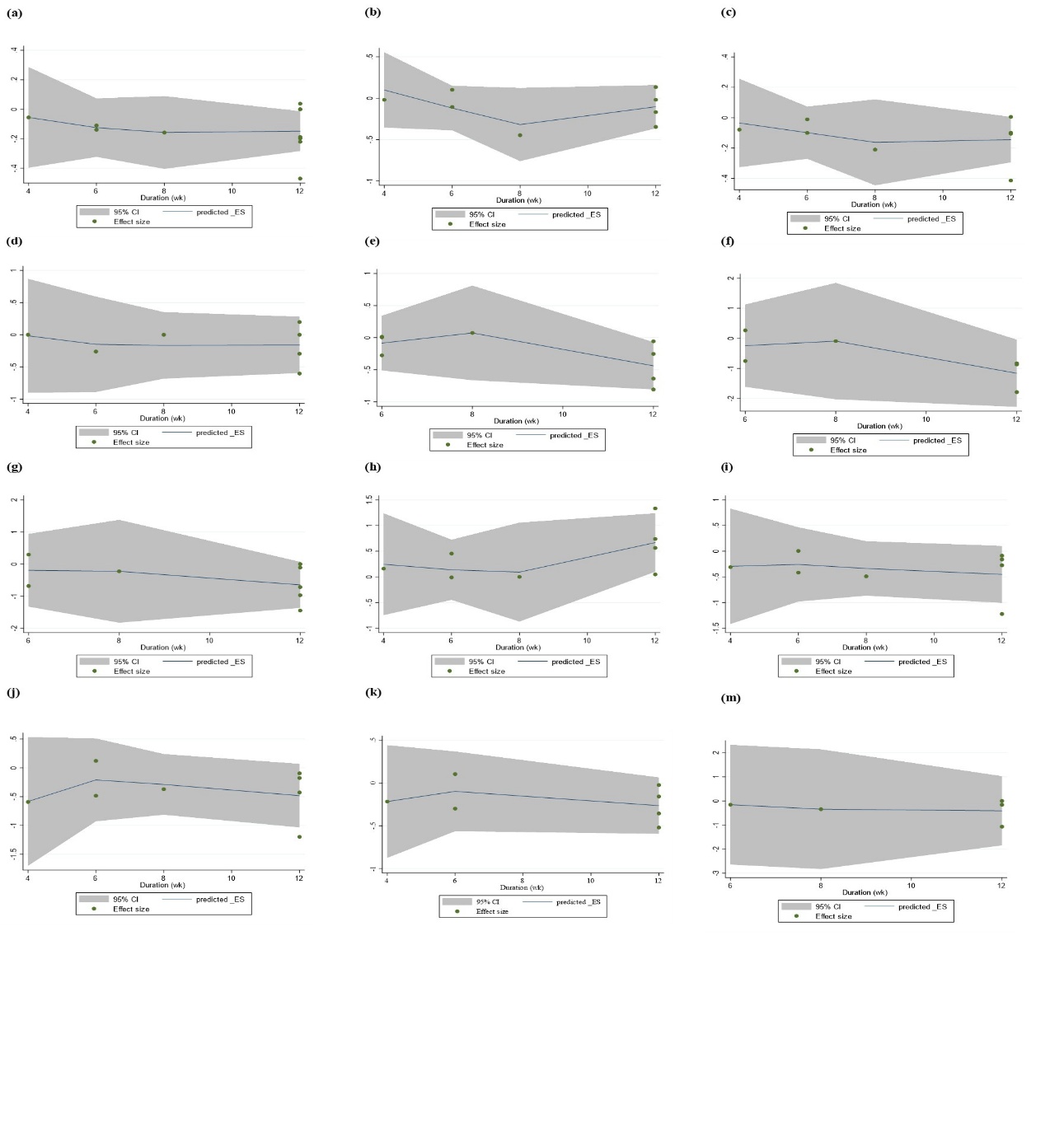


**Supplementary Figure 4.** Non-linear dose-response relations between duration of intervention and cardiovascular disease outcomes (a: BMI, b: WC, C: Weight, d: WHR, e: FBG, f: HOMA-IR, g: Serum Insulin, h: HDL, i: LDL, j: TC, k:TG, m: hsCRP)

# **Supplemental** **References**

1. Sabzghabaee AM, Kelishadi R, Golshiri K, Ghannadi A, Badri S. Clinical Effects of Rhus coriaria Fruits on Dyslipidemia308 in Adolescents: a Triple-blinded Randomized Placebo-controlled Trial. Med Arch. 2014;68(5):308-12.

2. Ardalani H, Moghadam MH, Rahimi R, Soltani J, Mozayanimonfared A, Moradi M, et al. Sumac as a novel adjunctive treatment in hypertension: a randomized, double-blind, placebo-controlled clinical trial. Rsc Advances. 2016;6(14):11507-12.

3. Hajmohammadi Z, Shams M, Zibainejad MJ, Nimrouzi M, Fardidi P, Heydari M. Efficacy and safety of Rhus coriaria in patients with hyperlipidemia: A randomized controlled trial. Planta Medica. 2016;82.

4. Alghadir AH, Gabr SA. Efficacy of Rhus coriaria (sumac) juice in reducing muscle pain during aerobic exercise. Physiol Int. 2016;103(2):231-42.

5. Rouhi-Boroujeni H, Mosharraf S, Gharipour M, Asadi-Samani M, Rouhi-Boroujeni H. Anti-hyperelipidemic effects of sumac (Rhus coriaria L.): Can sumac strengthen anti-hyperlipidemic effect of statins? Der Pharmacia Lettre. 2016;8(3):143-7.

6. Azali K. Effects of 10-weeks aerobic training with Rhus coriaria. L supplementation on TAC, insulin resistance and anthropometric indices in women with type 2 diabetes. Complementary Medicine Journal. 2017;7(1):1805-15.

7. Niknafs A, Rezvanfar M, Kamalinejad M, Latifi SA, Almasi-Hashiani A, Salehi M. The Effect of a Persian Herbal Medicine Compound on the Lipid Profiles of Patients with Dyslipidemia: A Randomized Double-Blind Placebo-Controlled Clinical Trial. Evid Based Complement Alternat Med. 2021;2021:6631963.

8. Kwak JH, Lee HJ, Jeong ST, Lee JY, Lee M, Paik JK. Effect of fermented Rhus verniciflua stokes extract on liver function parameters in healthy Korean adults: a double-blind randomized controlled trial. Trials. 2021;22(1):830.

9. Amirsasan R, Zakeri N, Gargari BP, Alizadeh S. The effect of anti-inflammatory properties of sumac powder (Rhus coriaria L.) along with ferrous sulfate on blood markers and oxidative stress of young athletic girls with non-anemic iron deficiency (NAID). Sport Sciences for Health. 2024;20(3):821-9.

10. Bagheri AR, Akbari H, Jafari MM, Rahmatpanah K, Jamshidi S, Momenzadeh F. COMPARISON OF THE EFFECT OF LIPEXAN HERBAL MEDICINE PRODUCT WITH PLACEBO AND GEMFIBROZIL ON BLOOD LIPID INDICES. New Armenian medical journal. 2024;18(1):121‐8.

11. Nadjarzadeh A, Fattahi MR, Moghtaderi F, Vahidi A, Hajiahmadi S, Mohsenpour MA. The Effect of Sumac Powder (Rhus Coriaria L) on Homocysteine and High-Sensitivity C-Reactive Protein in Patients with Type 2 Diabetes Mellitus. International Journal of Nutrition Sciences. 2024;9(1):71-9.
